# Components of the Clp complex are required for sporangium formation and dehiscence in *Actinoplanes missouriensis*

**DOI:** 10.1128/jb.00308-26

**Published:** 2026-09-01

**Authors:** Ryota Suzuki, Manato Maruko, Remi Suzuki, Takeaki Tezuka, Yasuo Ohnishi

**Affiliations:** 1 Department of Biotechnology, Graduate School of Agricultural and Life Sciences, The University of Tokyo 13143, Bunkyo-ku, Tokyo, Japan; 2 Collaborative Research Institute for Innovative Microbiology, The University of Tokyo 13143, Bunkyo-ku, Tokyo, Japan; University of Massachusetts Chan Medical School, Worcester, Massachusetts, USA

**Keywords:** *Actinoplanes missouriensis*, ATPase, caseinolytic protease (Clp), sporangium, spore release

## Abstract

The actinomycete *Actinoplanes missouriensis* forms terminal sporangia that contain a few hundred spores. When immersed in water, the sporangium opens to release spores, which start swimming using flagella, via a process referred to as sporangium dehiscence. In this study, we conducted a functional analysis of genes encoding the components of the caseinolytic protease (Clp) complex, which comprises ATPase (chaperone) subunits (ClpX and ClpC) and proteolytic subunits (ClpP1–4). The *clpX* null mutant (Δ*clpX*) strain formed sporangia smaller than the wild-type strain. The small sporangia scarcely opened under conditions that induce sporangium dehiscence in the wild-type strain. Consistently, the number of released spores was three orders of magnitude lower in the Δ*clpX* strain than in the wild-type strain. S1 nuclease mapping determined two transcriptional start points of *clpX* (TSS_U_ and TSS_D_ for upstream and downstream, respectively). A housekeeping sigma factor-dependent promoter and a FliA-family sigma factor-dependent promoter were found upstream of TSS_U_ and TSS_D_, respectively. A gene complementation test showed that apparently normal sporangium formation in the Δ*clpX* strain was restored by the introduction of *clpX* with either of the two promoters, whereas both promoters were required for sporangium dehiscence. Meanwhile, mutant strains that produced ClpC with T30S or I33F/L34F replacements, which presumably reduced their substrate-binding activity, produced sporangia with irregular shapes. Furthermore, gene disruption experiments of four putative proteolytic subunit genes indicated that *clpP3* is conditionally involved in sporangium dehiscence. We concluded that sporangium formation and dehiscence are regulated at the post-translational level via proteolysis by Clp complexes in *A. missouriensis*.

**IMPORTANCE:**

*Actinoplanes missouriensis* has a complex life cycle, in which the sporangium containing a few hundred flagellated spores is the most characteristic structure. Spores are released from sporangia via a process called sporangium dehiscence. We have revealed that sporangium formation and dehiscence are regulated by several transcriptional regulators; however, post-transcriptional regulation of sporangium formation and dehiscence remains unknown. In the present study, we revealed that two ATPase components of the Clp complex, ClpC and ClpX, and a proteolytic component, ClpP3, are involved in sporangium formation and/or dehiscence. This study indicates that in addition to transcriptional regulation, proteolysis regulated by Clp complexes is another crucial factor in the morphological development of *A. missouriensis*.

## INTRODUCTION

Caseinolytic protease (Clp) is a multimeric system that performs essential functions in maintaining protein homeostasis and regulatory proteolysis. This complex comprises an ATPase component known as Clp-ATPase and a proteolytic core referred to as ClpP (1). Clp-ATPases belong to the AAA+ (ATPase associated with diverse cellular activities) superfamily and form hexameric rings. The AAA+ superfamily is characterized by at least one AAA+ module, comprising a core nucleotide-binding domain and a smaller α-helical domain. This superfamily is categorized into two classes: class 1, which includes two AAA+ modules, and class 2, which contains a single AAA+ module (2). ClpA and ClpC are part of class 1, while ClpX belongs to class 2 ATPases. ClpC features an N-terminal domain followed by a nucleotide-binding domain (NBD-1), a middle domain (M domain), and a second nucleotide-binding domain (NBD-2). Although ClpA and ClpC exhibit similar domain structures, ClpA lacks the M domain (3). The nucleotide-binding domain found in ClpX is analogous to NBD-2 in class 1 proteins (3). ClpP functions as a self-compartmentalized serine protease, forming a barrel-like structure with two stacked heptameric rings. Within the rings, the catalytic triad of ClpP is enclosed in an internal chamber dedicated to the degradation of the substrate. To hydrolyze substrate proteins, ClpP partners with Clp-ATPases, which unfold and transport substrates into the degradation chamber using ATP (4). Because the proteolytic activity of ClpP has low sequence preferences, substrate specificity is conferred by cognate Clp-ATPases (5). In several cases, the Clp complex is regulated by adaptor proteins, such as ClpS in *Escherichia coli* and MecA in *Staphylococcus aureus* (6). ClpS in *E. coli* controls the selection of substrates by restricting or enhancing substrate recognition (7). MecA in *S. aureus* converts ClpC from an inactive complex to an active hexamer (8, 9).

Numerous bacterial species, including *E. coli*, *Bacillus subtilis*, *S. aureus*, and *Streptococcus pneumoniae*, contain a single ClpP homolog, resulting in the formation of homotetradecameric ClpP rings (10–13). Other bacteria, such as mycobacteria, chlamydia, listeria, clostridia, and pseudomonads, have two ClpP isoforms: ClpP1 and ClpP2. These microorganisms can create homotetradecameric rings from either ClpP1 or ClpP2 or heterotetradecameric rings that incorporate both isoforms (14–18). In contrast, the genus *Streptomyces* exhibits a more intricate Clp system. In *Streptomyces lividans*, the regulation of five *clpP* genes is achieved through multiple negative-feedback mechanisms. In this species, the transcriptional activator ClgR stimulates the expression of the *clpP1–clpP2* operon, while another activator, PopR, enhances the transcription of the *clpP3–clpP4* operon. Because ClpP1 and ClpP2 are responsible for degrading ClgR and PopR, the expression of the *clpP3–clpP4* operon remains inactive when ClpP1 and ClpP2 are produced (19–21).

Regulated proteolysis by the Clp complex is pivotal for survival under stress conditions because damaged proteins are recognized as substrates of the Clp complex (22). The Clp system manages various stress response strategies, such as inducing heat-shock chaperones, promoting natural competence, and facilitating motility and sporulation (5, 23). In *Salmonella* and *E. coli*, the ClpX–ClpP complex influences flagellar synthesis by suppressing the expression of flagellar genes through the post-translational degradation of the FlhD–FlhC master regulator complex (24–26). Accessory proteins, such as YdiV and FliT, enhance the proteolysis of FlhD–FlhC by releasing the regulator complex from the DNA (26, 27). In *B. subtilis*, a *clpP* deletion (Δ*clpP*) mutant has a non-motile filamentous morphology and displays a deficiency in sporulation and competence for DNA uptake (28). In *Streptomyces coelicolor* A3(2) and *S. lividans*, the Clp complex has been reported to play an essential role in aerial mycelium formation (29). Although the function of ClpP is not essential for survival in *E. coli* and *B. subtilis* (23, 25), at least one functional copy of either *clpP1–clpP2* or *clpP3–clpP4* is essential for the viability of *S. lividans*, suggesting that the Clp complex plays a fundamental role and that some functional redundancy exists among ClpP homologs (19). In *B. subtilis*, both ClpC and ClpP are essential for growth at high temperatures (28, 30).

*Actinoplanes missouriensis* is a representative species of the genus *Actinoplanes* (31). *A. missouriensis* grows as branched substrate mycelia during the vegetative phase and develops globose or subglobose terminal sporangia that emerge from the substrate mycelia through short sporangiophores. When exposed to dry conditions, it generates small sporangium-like structures by growing on humic acid-trace element (HAT) agar at 30°C for a period of 2 or 3 days. Fully developed sporangia appear after 5–7 days of incubation (31–34). Each sporangium houses several hundred spores encased within an intrasporangial matrix, with the sporangium envelope encircling the spores and the matrix on the sporangium surface (35, 36). Upon contact with water, the sporangium envelope disintegrates, allowing spores to be released into the surrounding environment. This phenomenon is referred to as sporangium dehiscence (34, 37–39). In a laboratory setting, sporangium dehiscence can be triggered by applying 25 mM NH_4_HCO_3_ solution to sporangia that developed on HAT agar, followed by incubation of the agar plate at room temperature for 1 h. Alternatively, sporangium dehiscence can be initiated by immersing sporangia collected from the sporangium-forming agar surface in 25 mM histidine solution, with the suspension incubated at room temperature for 1 h. Under the latter condition, phase-contrast microscopy allows us to observe the dehiscence process. Immediately after being suspended in the histidine solution, the sporangium envelope appears phase-bright, gradually turning transparent before the spores are released. Once released, spores swim in water using flagella as zoospores and exhibit chemotactic behavior. In environments conducive to vegetative growth, zoospores cease swimming and begin to germinate (40–42).

**Fig 1:**
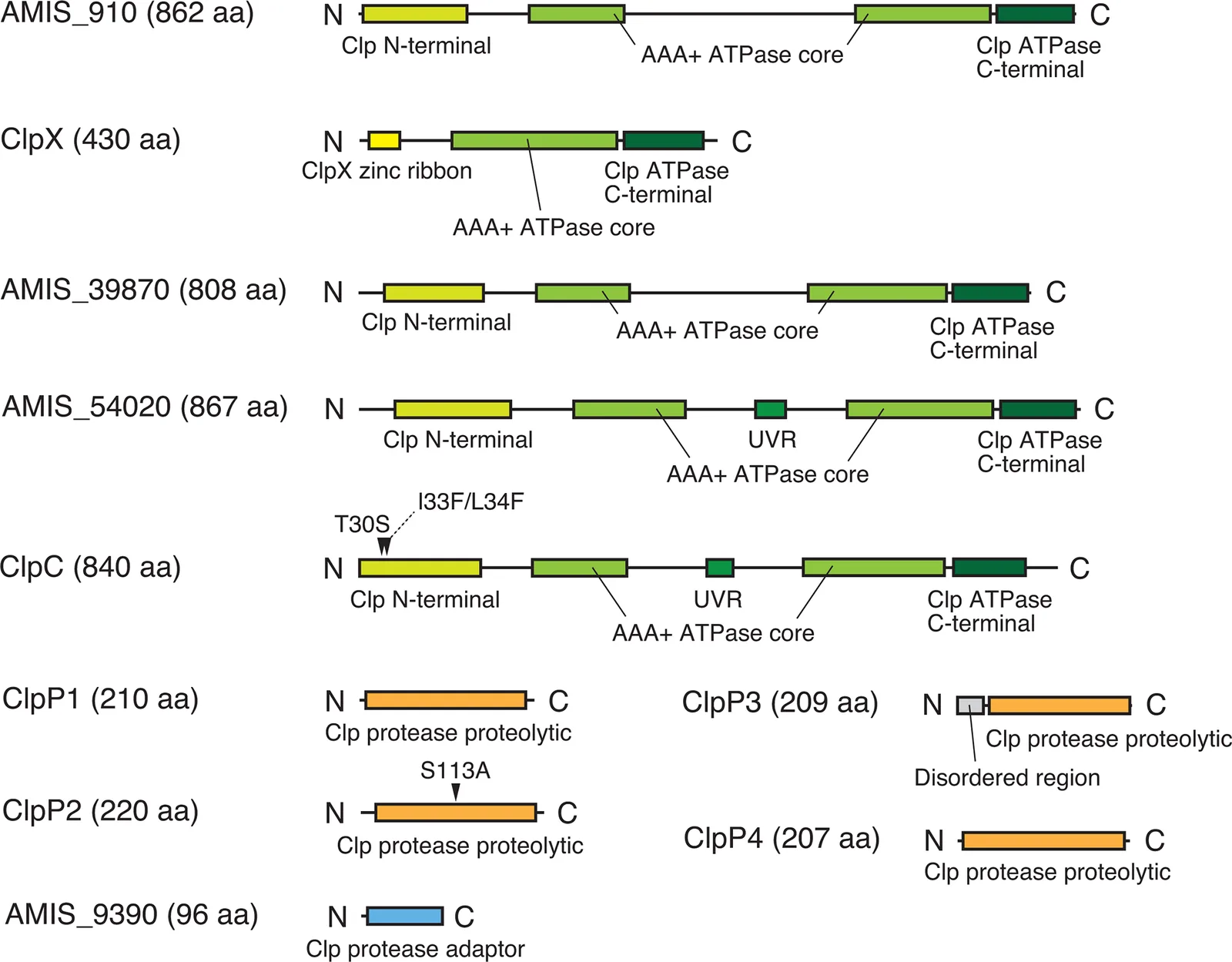
Domain organization of putative Clp components in *A. missouriensis*. Five Clp_ATPases (AMIS_910, ClpX, AMIS_39870, AMIS_54020, and ClpC), four proteolytic components (ClpP1, ClpP2, ClpP3, and ClpP4), and an adaptor protein (AMIS_9390) are shown. The locations of amino acid replacements in ClpC and ClpP2 are shown.

The complex developmental process in *A. missouriensis* is thought to rely on transcriptional regulation, protein modifications, and regulated proteolysis. Because one of the fundamental features of the Clp complex is the degradation of short-lived transcriptional regulators (1), the Clp complex is expected to be intimately involved in the morphological development of *A. missouriensis*. In the *A. missouriensis* genome, 10 genes are predicted to encode the Clp components (five Clp-ATPases, four ClpP proteases, and one Clp adaptor), suggesting the presence of relatively complex regulatory systems by the Clp complex. We recently reported that ClpC (AMIS_77690) is involved in the regulation of zoospore flagellar assembly by thioredoxin, TrxA, and its partner protein, PtxA (43). In the previous study, we proposed a working model in which a Clp complex containing ClpC hydrolyzes a flagellar secretion gate component, FliR, under flagellation-repressing conditions (43). Under flagellation-inducing conditions, the TrxA–PtxA complex possibly binds to ClpC in the Clp complex to inhibit its proteolytic activity (43). Point mutations were identified in the coding sequence of *clpC*, which replaced Thr-30 with Ser (T30S) and Ile-33/Leu-34 with Phe/Phe (I33F/L34F), in motile suppressor strains obtained from immotile *trxA* and *ptxA* null mutant strains (43). Thr-30, Ile-33, and Leu-34 are predicted to be located in the N-terminal domain of ClpC (Fig. 1; Fig. S1E). Because the N-terminal domain of Clp-ATPase is involved in substrate recognition and binding (44, 45), these ClpC variants are speculated to have reduced degradation activities against their substrate proteins. In the present study, through genetic and transcriptional analyses of putative Clp component genes, we propose that regulated proteolysis by the Clp complex is involved in sporangium formation and dehiscence in *A. missouriensis*.

## RESULTS

### *In silico* analysis of five Clp-ATPase, four ClpP, and one Clp adaptor protein candidates found in *A*. *missouriensis*

In the *A. missouriensis* chromosome, we found 10 genes encoding putative components of the Clp complex: five genes (*AMIS_910*, *AMIS_11250*, *AMIS_39870*, *AMIS_54020*, and *clpC*) encoding putative ATPase subunits, four genes (*AMIS_11230*, *AMIS_11240*, *AMIS_15950*, and *AMIS_72590*) encoding putative proteolytic subunits, and a gene (*AMIS_9390*) encoding a putative adaptor protein. A protein database search using InterPro ver. 106.0 (https://www.ebi.ac.uk/interpro/) revealed that AMIS_910, AMIS_39870, AMIS_54020, and ClpC, which are proteins of 862, 808, 867, and 840 amino acids, respectively, have a Clp N-terminal domain (accession number IPR004716), two ATPase AAA-type core domains (IPR003959), and a Clp ATPase C-terminal domain (IPR019489) (Fig. 1). The database search also revealed that AMIS_11250, a protein of 430 amino acids, harbors a ClpX-like zinc ribbon domain (IPR059188), an ATPase AAA-type core domain, and a Clp ATPase C-terminal domain (Fig. 1). Thus, AMIS_910, AMIS_39870, AMIS_54020, and ClpC are class 1 members of the AAA+ superfamily, whereas AMIS_11250 is a class 2 protein. We also predicted the structures of the five Clp_ATPases using ColabFold (https://colab.research.google.com/github/sokrypton/ColabFold/blob/main/AlphaFold2.ipynb) (46, 47). In the predicted structures of AMIS_910, AMIS_39870, AMIS_54020, and ClpC, a predicted M domain, which was not found in the crystal structure of ClpA from *E. coli* (48), was found within the NBD-1 domain (Fig. S1). Based on these *in silico* analyses, we considered AMIS_910, AMIS_39870, AMIS_54020, and ClpC to be ClpC-type ATPases and AMIS_11250 to be a ClpX-type protein. Hereafter, we named AMIS_11250 ClpX. We BLAST-searched the NCBI genome database (https://www.ncbi.nlm.nih.gov/genome/) and found that AMIS_910, AMIS_54020, ClpC, and ClpX homologs are highly conserved in *Actinoplanes* species; AMIS_910 and ClpX homologs were found in all of the 28 *Actinoplanes* species whose genome sequences have been registered in the database, including *A. missouriensis*, while AMIS_54020 and ClpC homologs were found in 25 *Actinoplanes* bacteria of the 28 species (Fig. S2A). Because 19 species that were previously classified into the genus *Actinoplanes* have been reclassified into two phylogenetically related genera, *Paractinoplanes* and *Winogradskya*, the number of registered *Actinoplanes* genomes is smaller than that of similar analyses in our previous studies (42, 43). It should be noted that AMIS_39870 is located near the AMIS_54020 homologs in this phylogenetic tree because AMIS_39870 and AMIS_54020 share 58% sequence identity (73% similarity) (Fig. S2A).

The database search using InterPro also revealed that AMIS_11230, AMIS_11240, AMIS_15950, and AMIS_72590, which are proteins of 210, 220, 209, and 207 amino acids, respectively, harbor an ATP-dependent Clp protease proteolytic subunit domain (IPR001907) (Fig. 1). Hereafter, we named the four proteolytic components (AMIS_11230, AMIS_11240, AMIS_15950, and AMIS_72590) ClpP1, ClpP2, ClpP3, and ClpP4, respectively. Amino acid sequence alignment showed that ClpP1, ClpP2, and ClpP4 share high sequence similarities with each other (40%–61% identities; Fig. S3A and B). In contrast, ClpP3 shares lower sequence similarity with ClpP1, ClpP2, and ClpP4 (22%–26% identities; Fig. S3A and B). We found that ClpP1, ClpP2, and ClpP4 homologs were conserved in all of the 28 *Actinoplanes* species whose genome sequences have been registered in the database, including *A. missouriensis* (Fig. S2B). In contrast, ClpP3 homologs were found in 19 *Actinoplanes* bacteria of the 28 species (Fig. S2B).

The crystal structure of ClpP from *E. coli* (*Ec*ClpP) revealed that the ClpP oligomer has three major solvent-exposed surfaces: the exterior sides, interior proteolytic chamber, and exterior end-on (10). In the Clp complex, the ATPase component binds to the exterior end-on surface of ClpP, which has a sprocket-like appearance (10, 49, 50). This surface is hydrophobic and comprises solvent-exposed aromatic residues, including Tyr-60, Tyr-62, and Phe-82 in *Ec*ClpP (Fig. S3A). The conserved surface of the exterior end-on stabilizes the ClpP-ATPase component complex. Amino acid sequence alignment of *Ec*ClpP and the four ClpP proteins from *A. missouriensis* showed that at least one Tyr or Phe residue is conserved in ClpP1, ClpP2, and ClpP4 at the residues corresponding to Tyr-60, Tyr-62, and Phe-82 of *Ec*ClpP (one Phe residue in ClpP1 and two Tyr residues in ClpP2 and ClpP4; Fig. S3A). In contrast, no aromatic residue was found in ClpP3 around the residues corresponding to the three *Ec*ClpP residues (Fig. S3A). Furthermore, the sequence alignment revealed that only ClpP3 does not possess a catalytic triad in its amino acid sequence (Fig. S3A). Therefore, these *in silico* analyses suggest that ClpP3 is catalytically inactive and not involved in the interaction with the ATPase component. It should be noted that the N-terminal region of ClpP3 was predicted to be disordered by MobiDB-lite (http://old.protein.bio.unipd.it/mobidblite/) (51) (Fig. 1).

The database search predicted that AMIS_9390 (96 amino acids) harbors an ATP-dependent Clp protease adaptor protein ClpS domain (IPR022935) (Fig. 1). In the MecA/ClpC/ClpP complex of *S. aureus*, the adaptor protein MecA interacts with the N-terminal and M domains of ClpC (9).

### ClpX is required for normal sporangium formation in *A*. *missouriensis*

**Fig 2:**
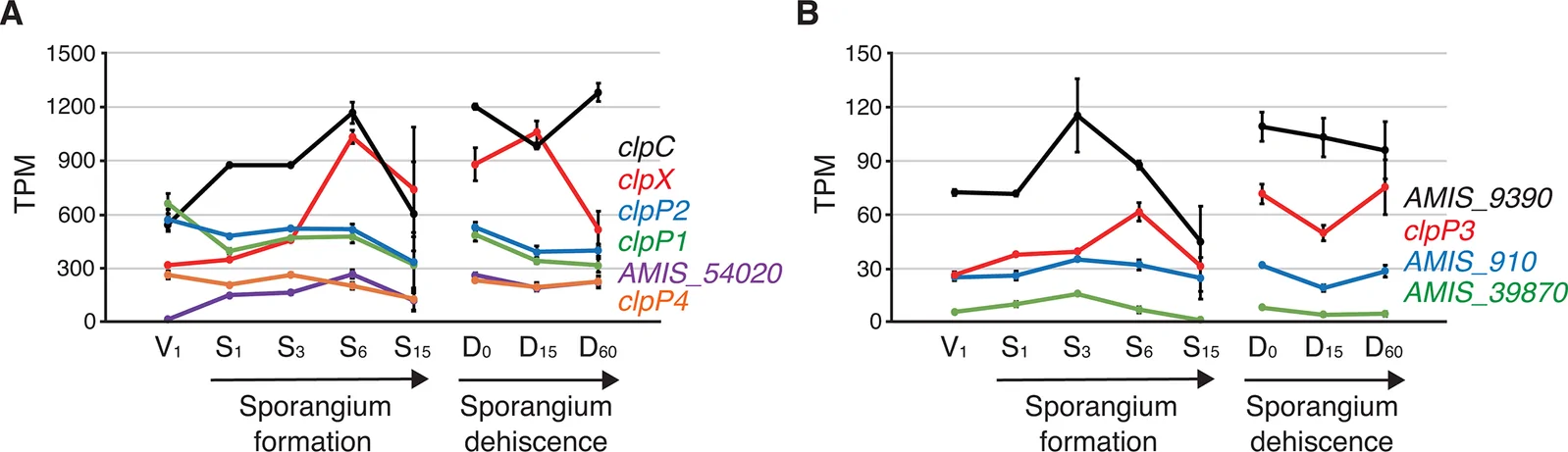
Transcript levels of 10 genes encoding Clp components. (**A**) The *clpX*, *AMIS_54020*, *clpC*, *clpP1*, *clpP2*, and *clpP4* genes. (**B**) The *AMIS_910*, *AMIS_9390*, *AMIS_39870*, and *clpP3* genes. RNA samples for RNA sequencing analysis were prepared from substrate hyphae grown on YBNM agar for 1 day (V_1_), substrate hyphae or mixtures of substrate hyphae and sporangia grown on HAT agar for 1, 3, 6, and 15 days (S_1_, S_3_, S_6_, and S_15_, respectively), and sporangia (including some substrate hyphae) incubated in 25 mM histidine solution to induce sporangium dehiscence for 0, 15, and 60 min (D_0_, D_15_, and D_60_, respectively). The average number of transcripts per million mapped reads (TPM) values ± standard errors from three biological replicates are shown.

In an earlier investigation, we performed RNA sequencing at different stages of the *A. missouriensis* life cycle (39). For the analysis, RNA samples representing the vegetative phase were collected from substrate hyphae cultivated on yeast extract-beef extract-NZ amine-maltose monohydrate (YBNM) agar for 1 day, with three replicates. To analyze sporangium formation, RNA was extracted from mycelia and/or sporangia grown on HAT agar for 1, 3, 6, and 15 days, with triplicate samples at each time point. For sporangium dehiscence, RNA samples were obtained from sporangia (including some substrate hyphae) that were immersed and incubated in 25 mM histidine solution for 0, 15, and 60 min in triplicate for each time point. This analysis allowed us to compare the transcriptional profiles of genes encoding ATPase, proteolytic, and adaptor components of the Clp complex (Fig. 2). Of the 10 genes examined, we focused on *clpX* because of its significantly elevated transcript levels during the late phase of sporangium formation (day 6 on HAT agar) (Fig. 2A).

**Fig 3:**
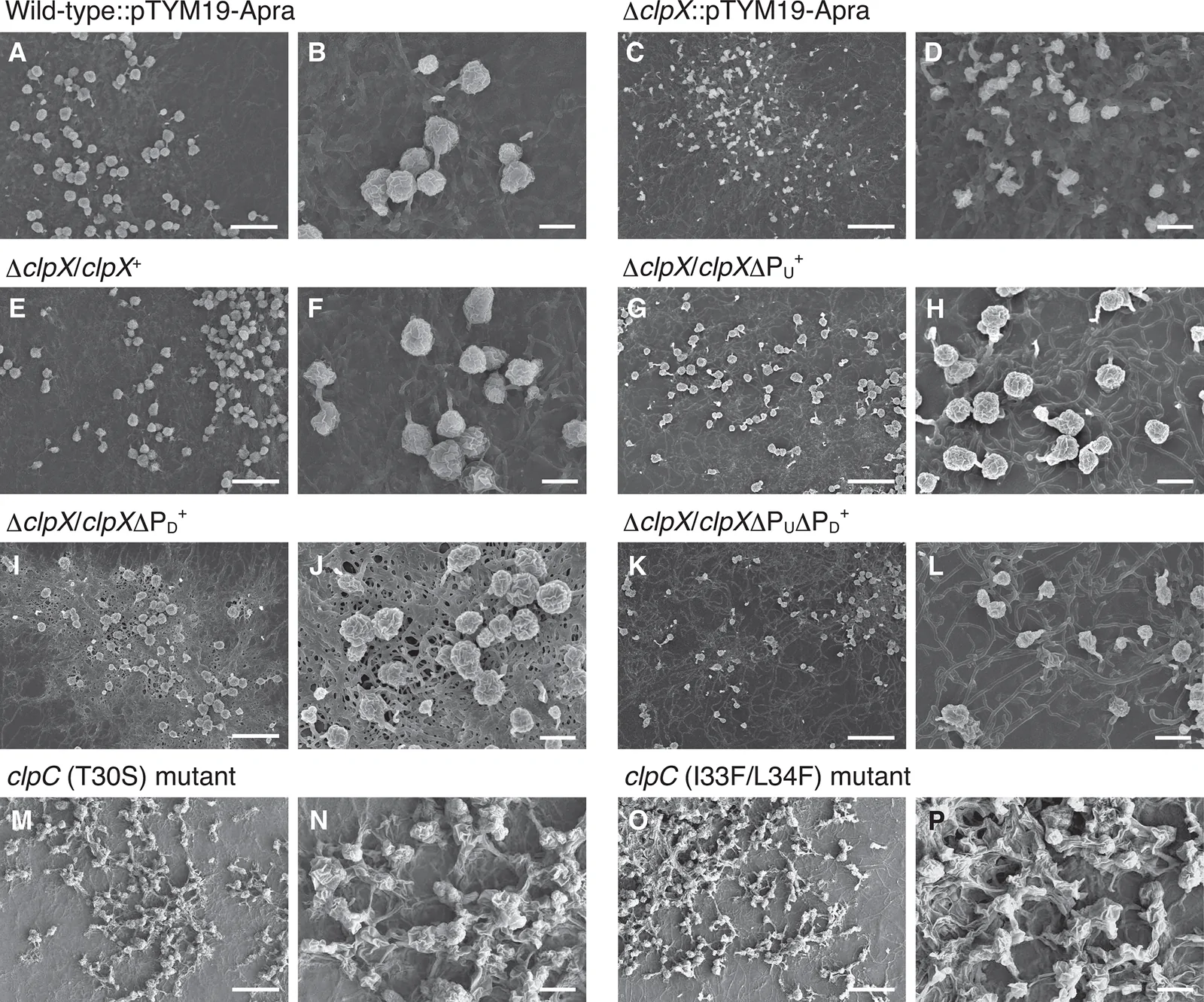
SEM analysis of sporangia and mycelia produced on HAT agar after 7 days of cultivation. (**A and B**) Wild-type strain harboring the empty vector pTYM19-Apra. (**C and D**) Δ*clpX* strain harboring pTYM19-Apra. (**E and F**) Δ*clpX* strain harboring the *clpX* complementation plasmid. (**G and H**) Δ*clpX* strain harboring the *clpX*ΔP_U_ complementation plasmid. (**I and J**) Δ*clpX* strain harboring the *clpX*ΔP_D_ complementation plasmid. (**K and L**) Δ*clpX* strain harboring the *clpX*ΔP_U_ΔP_D_ complementation plasmid. (**M and N**) *clpC* (T30S) mutant strain. (**O and P**) *clpC* (I33F/L34F) mutant strain. Bars, 20 μm (**A, C, E, G, I, K, M, and O**) or 5 μm (**B, D, F, H, J, L, N, and P**).

To examine the *in vivo* functions, we generated a *clpX* null mutant (Δ*clpX*) strain. No differences were observed between the wild-type and Δ*clpX* strains cultivated on YBNM and HAT agars by macroscopic observation. Thus, we observed sporangium formation in both strains grown on HAT agar at 30°C for 7 days using scanning electron microscopy (SEM). In the following phenotypic analysis, the wild-type and Δ*clpX* strains harbored the chromosome-integrating vector pTYM19-Apra (52, 53) because these pTYM19-Apra-integrated strains were used as control strains for gene complementation tests, as described below. The wild-type strain produced normal globose or subglobose sporangia (Fig. 3A and B). The Δ*clpX* strain produced sporangia, but they were smaller than the wild-type sporangia (Fig. 3C and D), indicating that ClpX is required for normal sporangium formation. In a gene complementation test, the introduction of *clpX* with its own promoter into the Δ*clpX* strain resulted in normal sporangium formation (Fig. 3E and F).

**Fig 4:**
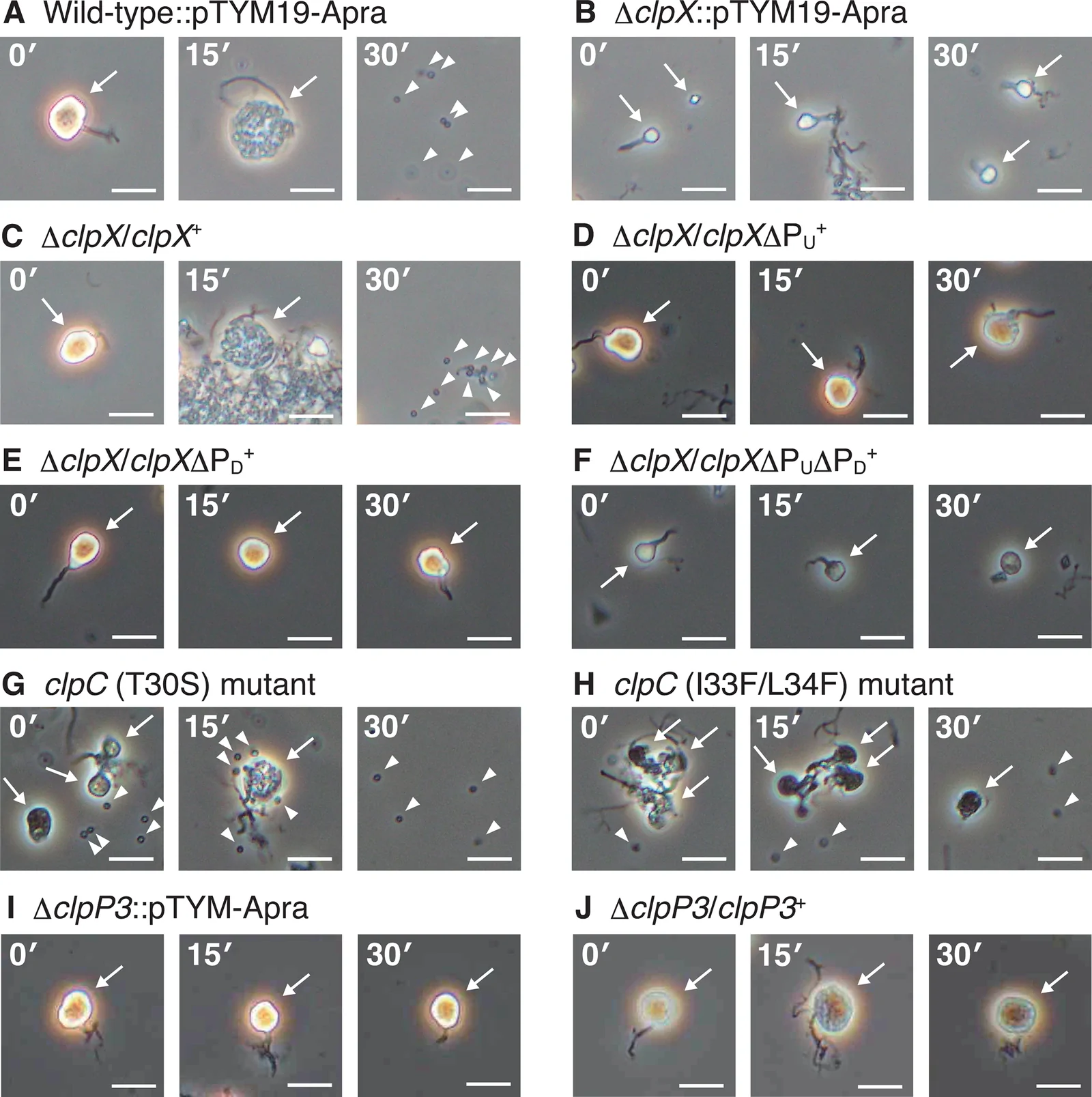
Observation of sporangium dehiscence by phase-contrast microscopy. Sporangia produced on HAT agar were harvested and suspended in 25 mM histidine solution to induce sporangium dehiscence. Micrographs of the wild-type strain harboring pTYM19-Apra (**A**), Δ*clpX* strain harboring pTYM19-Apra (**B**), Δ*clpX* strain harboring the *clpX* complementation plasmid (**C**), Δ*clpX* strain harboring the *clpX*ΔP_U_ complementation plasmid (**D**), Δ*clpX* strain harboring the *clpX*ΔP_D_ complementation plasmid (**E**), Δ*clpX* strain harboring the *clpX*ΔP_U_ΔP_D_ complementation plasmid (**F**), *clpC* (T30S) mutant strain (**G**), *clpC* (I33F/L34F) mutant strain (**H**), Δ*clpP3* strain harboring pTYM19-Apra (**I**), and Δ*clpP3* strain harboring the *clpP3* complementation plasmid (**J**) are shown. The left, middle, and right panels of each data set were taken immediately, 15 min, and 30 min, respectively, after suspension. Immediately after suspension, the sporangium envelope was phase-bright (**A, C, D, E, I, and J**). The sporangium envelope gradually became transparent before the release of spores (**A and C**). Sporangia (including sporangia whose envelope became transparent and small sporangia) and released spores are indicated by arrows and arrowheads, respectively. Bars, 10 μm.

Next, we induced sporangium dehiscence by suspending sporangia harvested from the sporangium-forming agar surface in 25 mM histidine solution. Wild-type sporangia opened and released spores normally when observed using phase-contrast microscopy (Fig. 4A). In contrast, sporangium dehiscence was not observed in the Δ*clpX* strain (Fig. 4B), suggesting that the small sporangia of the Δ*clpX* strain were not sufficiently mature to release spores under the tested conditions. The sporangia of the Δ*clpX* strain harboring the *clpX* complementation plasmid opened normally and released spores under the same conditions (Fig. 4C).

**Fig 5:**
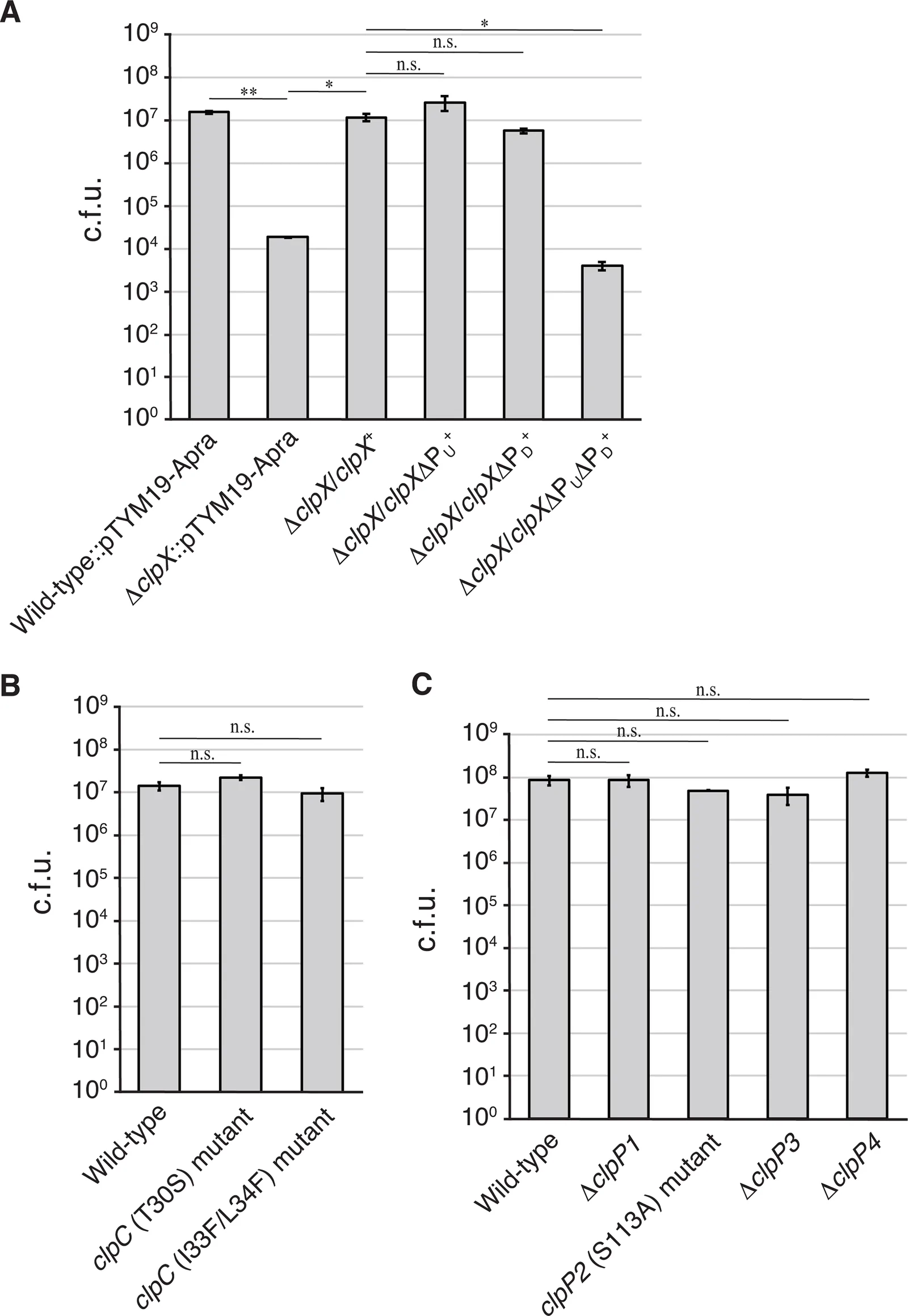
The number of spores released from the sporangia. Each strain was cultivated on HAT agar at 30°C for 7 days. Sporangium dehiscence was induced by pouring 25 mM NH_4_HCO_3_ solution on sporangium-forming HAT agar, followed by incubation of the agar plate at room temperature for 1 h. The solution was retrieved from the agar surface and filtered through a 5-μm membrane filter to eliminate the mycelia and sporangia. A portion of the zoospore suspension was cultivated on YBNM agar at 30°C for 2 days. The number of colonies was counted to estimate the colony-forming unit (c.f.u.) value of the sample. The values are the mean ± standard error of three biological replicates. Differences were analyzed using Student’s *t*-test. * and ** mean *P* < 0.01 and *P* < 0.001, respectively. “n.s.” means no significant difference (*P* > 0.05). (**A**) Wild-type and Δ*clpX* strains, both of which harbored pTYM19-Apra, and Δ*clpX* strains harboring the *clpX*, *clpX*ΔP_U_, *clpX*ΔP_D_, and *clpX*ΔP_U_ΔP_D_ complementation plasmids. (**B**) Wild-type, *clpC* (T30S) mutant, and *clpC* (I33F/L34F) mutant strains. (**C**) Wild-type, Δ*clpP1*, *clpP2* (S113A) mutant, Δ*clpP3*, and Δ*clpP4* strains.

We quantified the spores released from sporangia of the wild-type and Δ*clpX* strains by pouring 25 mM NH_4_HCO_3_ solution onto the sporangium-forming HAT agar plates, followed by counting the colonies formed on YBNM agar after incubation at 30°C for 2 days. In this experiment, all colonies on YBNM agar were formed from the spores released from sporangia because the zoospore suspension retrieved from the HAT agar surface was filtered through a 5-μm membrane filter to eliminate mycelia and sporangia. Approximately 10^7^ spores were released from the wild-type sporangia formed on one HAT agar plate (Fig. 5A). In contrast, the Δ*clpX* sporangia released only 10^4^ spores per HAT agar plate (Fig. 5A), which is consistent with the abnormal sporangium formation and dehiscence observed in the Δ*clpX* strain. In a gene complementation test, the number of spores released from Δ*clpX* sporangia was restored by introducing the *clpX* complementation plasmid (Fig. 5A).

### Transcriptional regulation of *clpX*

**Fig 6:**
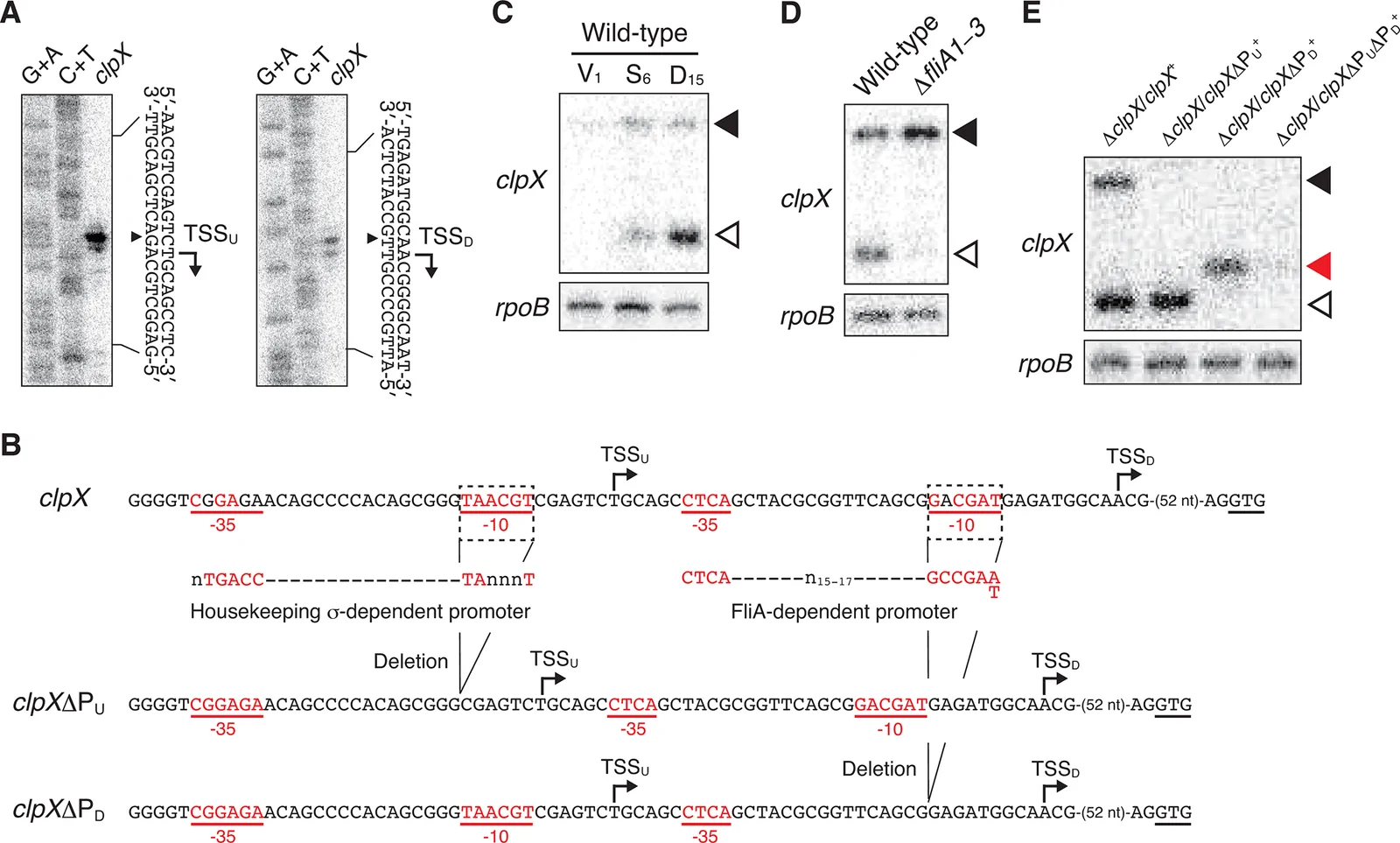
Transcriptional regulation of *clpX*. (**A**) Transcriptional start points of *clpX* determined by high-resolution S1 nuclease mapping. Arrowheads indicate the positions of S1 nuclease-protected fragments. The 5′-termini of mRNAs were assigned to the indicated positions because the fragments generated by the chemical sequencing reactions migrate 1.5 nucleotides further than the corresponding fragments generated by S1 nuclease digestion of the DNA-RNA hybrids (half a residue from the presence of the 3′-terminal phosphate group and one residue from the elimination of the 3′-terminal nucleotide). (**B**) Nucleotide sequences of the upstream regions from *clpX*, *clpX*ΔP_U_, and *clpX*ΔP_D_. Transcriptional start points are indicated by bent arrows. Putative housekeeping sigma factor- and FliA-family sigma factor-dependent promoter elements are indicated by red letters. Start codons are underlined. (**C**) Transcriptional analysis of *clpX* in the wild-type strain by S1 nuclease mapping. RNA samples were extracted from the vegetative mycelia cultivated in PYM liquid broth at 30°C for 1 day (V_1_), a mixture of substrate mycelia and sporangia grown on HAT agar at 30°C for 6 days (S_6_), and a mixture of substrate mycelia and sporangia suspended in 25 mM histidine solution and incubated with rotation for 15 min (D_15_) to induce sporangium dehiscence. (**D**) Transcriptional analysis of *clpX* in the wild-type and Δ*fliA1*Δ*fliA2*Δ*fliA3* (Δ*fliA1–3*) strains by S1 nuclease mapping. RNA samples were extracted from both strains grown on HAT agar at 30°C for 6 days. (**E**) Transcriptional analysis of *clpX* in the Δ*clpX* strains harboring the *clpX*, *clpX*ΔP_U_, *clpX*ΔP_D_, and *clpX*ΔP_U_ΔP_D_ complementation plasmids. RNA samples were extracted from each strain grown on HAT agar at 30°C for 6 days. (**C–E**) Transcripts of *rpoB*, which encodes the β subunit of RNA polymerase, were used as loading controls. The S1 nuclease-protected fragments from the upstream and downstream transcriptional start points are indicated by closed and open triangles, respectively. (**E**) The S1 nuclease-protected fragment detected in the Δ*clpX* strain harboring *clpX*ΔP_D_ is indicated by a red triangle, which corresponds to the endpoint of the deletion site in the FliA-family sigma factor-dependent promoter (32 nucleotides shorter than the fragment from the upstream transcriptional start point) due to the mismatch between the probe and transcripts.

We determined two transcriptional start points of *clpX* to be 98 and 57 nucleotides upstream from the start codon (referred to as TSS_U_ and TSS_D_, respectively, for upstream and downstream transcriptional start sites) by high-resolution S1 nuclease mapping (Fig. 6A). As reported in our previous study (54), three FliA-family sigma factors, FliA1, FliA2, and FliA3, are involved in sporangium formation, spore dormancy, and sporangium dehiscence in *A. missouriensis*. Sequence scanning revealed a putative promoter element that could be recognized by the housekeeping sigma factor (55) in the upstream region of TSS_U_, whereas a FliA-family sigma factor-dependent promoter element was found in the upstream region of TSS_D_ (Fig. 6B) (54). The locations of the elements are typical for the housekeeping sigma factor- and the FliA-family sigma factor-dependent promoters (54, 55).

We analyzed the transcript levels of *clpX* from two promoters in the wild-type strain by S1 nuclease mapping using RNA samples extracted from vegetative hyphae cultivated in peptone-yeast extract-magnesium (PYM) broth, a mixture of substrate mycelia and sporangia cultivated on HAT agar for 6 days, and sporangia (containing some substrate mycelia) incubated in 25 mM histidine solution for 15 min to induce sporangium dehiscence. The transcripts from both promoters were scarcely detected in the vegetative hyphae (Fig. 6C, lane V_1_). In contrast, transcripts from both promoters were detected at similar levels in a mixture of substrate mycelia and sporangia (Fig. 6C, lane S_6_). The transcript level from TSS_D_ increased in sporangia under the dehiscence-inducing condition, whereas the transcript from TSS_U_ was detected at a similar level to the mixture of substrate mycelia and sporangia under the same condition (Fig. 6C, lane D_15_).

We performed another transcriptional analysis to examine whether FliA-family sigma factors recognize the promoter element upstream of TSS_D_. In this experiment, we analyzed the transcript levels of *clpX* by S1 nuclease mapping using the wild-type strain and a mutant strain lacking *fliA1*, *fliA2*, and *fliA3* (Δ*fliA1*Δ*fliA2*Δ*fliA3*), which had been generated in our previous study (54). RNA samples were prepared from mixtures of substrate mycelia and sporangia grown on HAT agar for 6 days. The transcript from TSS_D_ was not detected in the Δ*fliA1*Δ*fliA2*Δ*fliA3* strain, while the transcript from TSS_U_ was detected at a similar level in the wild-type and Δ*fliA1*Δ*fliA2*Δ*fliA3* strains (Fig. 6D). This result demonstrated that at least one of the three FliA-family sigma factors is essential for the transcription of *clpX* from TSS_D_.

### Transcription of *clpX* from two promoters is conditionally required for sporangium dehiscence

To investigate whether the two *clpX* promoters are required for sporangium formation and dehiscence, we generated two mutated genes, *clpX*ΔP_U_ and *clpX*ΔP_D_, in which the −10 box motifs of the housekeeping sigma factor- and FliA-family sigma factor-dependent promoters, respectively, were deleted (Fig. 6B). Furthermore, we generated a mutated gene, *clpX*ΔP_U_ΔP_D_, in which the −10 motifs of both promoters were deleted. We introduced wild-type and three mutated (ΔP_U_, ΔP_D_, and ΔP_U_ΔP_D_) *clpX* gene constructs into the Δ*clpX* strain and confirmed that transcription from the mutated promoter elements was not detected by S1 nuclease mapping (Fig. 6E). In this experiment, there were sequence mismatches between the probe and the transcripts from the Δ*clpX* strains harboring *clpX*ΔP_D_ and *clpX*ΔP_U_ΔP_D_ because the probe prepared from the wild-type sequence was used for all strains. Thus, the digestion product by S1 nuclease detected in the Δ*clpX* strain harboring *clpX*ΔP_D_ corresponded to the endpoint of the deletion site in the FliA-family sigma factor-dependent promoter, not to the transcription start point from the housekeeping sigma factor-dependent promoter (Fig. 6E). In the Δ*clpX* strain harboring *clpX*ΔP_U_ΔP_D_, almost no digestion products were detected due to the deletions in both promoters (Fig. 6E). We then observed the sporangia and mycelia of these strains grown on HAT agar at 30°C for 7 days using SEM. As a result, the Δ*clpX* strains harboring *clpX*ΔP_U_ and *clpX*ΔP_D_ formed apparently normal sporangia, as observed in the Δ*clpX* strain harboring the wild-type *clpX* gene (Fig. 3E through J). In contrast, sporangia smaller than those of the wild-type strain were observed in the Δ*clpX* strain harboring *clpX*ΔP_U_ΔP_D_, as observed in the Δ*clpX* strain harboring the empty vector (Fig. 3K and L). These results showed that either of the two *clpX* promoters is sufficient for sporangium formation.

Based on SEM observations, we expected that the Δ*clpX* strains harboring *clpX*ΔP_U_ and *clpX*ΔP_D_ would form mature sporangia that could open to release spores under sporangium dehiscence-inducing conditions. Thus, we suspended the sporangia of these strains harvested from HAT agar in 25 mM histidine solution and observed sporangium dehiscence using phase-contrast microscopy. However, the normal-shaped sporangia of the Δ*clpX* strains harboring *clpX*ΔP_U_ and *clpX*ΔP_D_ did not open under the tested conditions (Fig. 4D and E). In this experiment, the small sporangium-like structures of the Δ*clpX* strain harboring *clpX*ΔP_U_ΔP_D_ did not open, as observed in the Δ*clpX* strain harboring the empty vector (Fig. 4F). As described above, the sporangia of the Δ*clpX* strain harboring the wild-type *clpX* gene normally opened and released spores under the same conditions (Fig. 4C). Therefore, these results suggest that both *clpX* promoters are required for sporangium dehiscence. It should be noted that the deficiency in sporangium dehiscence of the Δ*clpX* strains harboring *clpX*ΔP_U_ and *clpX*ΔP_D_ is conditional because similar numbers of spores were released under different sporangium dehiscence-inducing conditions, as described below.

We quantified the spores released from the sporangia of the Δ*clpX* strains harboring *clpX*ΔP_U_, *clpX*ΔP_D_, and *clpX*ΔP_U_ΔP_D_. In this experiment, a 25 mM NH_4_HCO_3_ solution was poured onto the sporangium-forming HAT agar plate, followed by incubation of the plate for 1 h to induce sporangium dehiscence. Approximately 10^7^ spores were released from the sporangia of the Δ*clpX* strains harboring *clpX*ΔP_U_ and *clpX*ΔP_D_ formed on one HAT agar plate, and these numbers of released spores were comparable to the number of spores released from the Δ*clpX* strain harboring the wild-type *clpX* gene (Fig. 5A). This result was inconsistent with the observation of sporangium dehiscence by phase-contrast microscopy (Fig. 4C through E). We assume that this inconsistency was derived from the differences in the conditions used in the two experiments. Therefore, the deficiency in sporangium dehiscence in the Δ*clpX* strains harboring *clpX*ΔP_U_ and *clpX*ΔP_D_ is conditional. The sporangia of the Δ*clpX* strain harboring *clpX*ΔP_U_ΔP_D_ released a much smaller number of spores, similar to the Δ*clpX* strain harboring the empty vector (Fig. 5A).

### Phenotypic analysis of *clpC* point mutant strains

As described in the Introduction, we reported that ClpC is involved in the regulation of zoospore flagellar assembly by TrxA and PtxA (43). In the previous study, we tried but failed to obtain a *clpC* null mutant strain, suggesting that the gene product is essential for growth (43). Thus, to further characterize the physiological functions of ClpC, the T30S and I33F/L34F mutations were introduced in *clpC* on the chromosome of the wild-type strain, generating the *clpC* (T30S) and *clpC* (I33F/L34F) mutant strains, respectively. We observed the *clpC* (T30S) and *clpC* (I33F/L34F) mutant strains grown on HAT agar at 30°C for 7 days using SEM. These mutant strains produced abnormal sporangia with squashed shapes (Fig. 3M through P). Although the mutant sporangia seemed to be forcibly squashed during the process of sample preparation for SEM analysis, as observed previously for a *tcrA* null mutant strain (56), this result indicated that ClpC is involved in the formation of normal sporangia. We induced sporangium dehiscence by suspending the mutant sporangia harvested from the sporangium-forming HAT agar surface in 25 mM histidine solution. In both mutants, the envelope of most sporangia was phase-dark, and some spores were released into the external environment immediately after suspension in histidine solution (Fig. 4G and H). We assume that the mutant sporangia are structurally fragile and that part of the sporangia collapsed under aquatic conditions, as observed previously for a spore surface protein (SspA)-deficient mutant (57). We quantified the spores released from sporangia of the wild-type strain and the *clpC* (T30S) and *clpC* (I33F/L34F) mutant strains by counting colonies formed on YBNM agar after incubation at 30°C for 2 days. In this experiment, 25 mM NH_4_HCO_3_ solution was poured onto the sporangium-forming HAT agar plate, followed by incubation of the plate for 1 h to induce sporangium dehiscence. The number of colonies formed on YBNM agar did not differ significantly between the wild-type and mutant strains (Fig. 5B). This result indicated that the wild-type and mutant strains produced similar numbers of viable spores. Based on these observations by SEM and phase-contrast microscopy, we speculate that the *clpC* (T30S) and *clpC* (I33F/L34F) mutant sporangia collapsed and released spores, possibly in addition to spore release through the normal dehiscence process.

### ClpP3 is conditionally required for sporangium dehiscence in *A*. *missouriensis*

We generated null mutant strains of *clpP1*, *clpP3*, and *clpP4* (Δ*clpP1*, Δ*clpP3*, and Δ*clpP4*, respectively). Notably, we also attempted to obtain a *clpP2* null mutant strain, but failed, suggesting that ClpP2 is essential for growth under the culture conditions used in this study. Thus, we introduced a mutation into the coding sequence of *clpP2*, which replaced Ser-113 of the catalytic triad with Ala (S113A; Fig. S3A). We successfully generated a *clpP2* (S113A) mutant strain, suggesting that the proteolytic activity of ClpP2 is not required for *A. missouriensis* viability.

No differences were observed among the wild-type, Δ*clpP1*, *clpP2* (S113A), Δ*clpP3*, and Δ*clpP4* strains cultivated on YBNM and HAT agar plates by macroscopic observation. We observed these strains grown on HAT agar at 30°C for 7 days using SEM. All strains produced apparently normal globose or subglobose sporangia (Fig. S4), indicating that the protease activity of each proteolytic subunit of the Clp complex is not required for sporangium formation. Notably, ClpP3 is expected to be catalytically inactive, as described above. Next, we examined sporangium dehiscence by phase-contrast microscopy. In this analysis, the Δ*clpP3* strain harbored the vector pTYM19-Apra because the empty vector-integrated strain was used as a control strain for the gene complementation test, as described below. The sporangia of the Δ*clpP1*, *clpP2* (S113A), and Δ*clpP4* strains opened and released spores similarly to the wild-type sporangia when suspended in 25 mM histidine solution (Fig. S5). In contrast, the sporangia of the Δ*clpP3* strain did not open under the same conditions (Fig. 4I), indicating that ClpP3 is required for sporangium dehiscence. Therefore, we induced the release of spores by pouring 25 mM NH_4_HCO_3_ solution on the sporangium-forming HAT agar and quantified the spores as described above. Approximately 10^7^–10^8^ spores were released from the sporangia of all strains formed on one HAT agar plate (Fig. 5C). In a gene complementation test, the introduction of *clpP3* with its own promoter into the Δ*clpP3* strain did not result in spore release from sporangia when suspended in 25 mM histidine solution, although the sporangia gradually became phase-dark (Fig. 4J). The incomplete recovery of phenotypic changes may be due to the difference in the *clpP3* expression levels between the wild-type strain and the Δ*clpP3* strain harboring the *clpP3* complementation plasmid. In conclusion, no phenotypic changes were observed in the Δ*clpP1*, *clpP2* (S113A), and Δ*clpP4* strains, but the Δ*clpP3* strain showed a conditional deficiency in sporangium dehiscence, similar to the Δ*clpX* strains harboring *clpX*ΔP_U_ and *clpX*ΔP_D_, as described above.

## DISCUSSION

In this study, we investigated the involvement of Clp complex components in the morphological development of *A. missouriensis*. The phenotypic analysis of the Δ*clpX*, *clpC* (T30S), and *clpC* (I33F/L34F) strains clearly demonstrated that ClpX and ClpC are essential for normal sporangium formation. We assume that the Clp complex containing ClpX or ClpC as an ATPase component hydrolyzes proteins that repress the normal progression of sporangium formation. In a previous study, we introduced the T30S and I33F/L34F mutations in *clpC* of immotile *trxA* and *ptxA* null mutant strains by allelic replacement, demonstrating that these mutations restored the mobility of both mutant strains (43). Considering the phenotypic changes in the *clpC* (T30S) and *clpC* (I33F/L34F) strains in the present study, the ClpC-containing Clp complex seems to be involved in sporangium formation and dehiscence besides flagellar formation. The Δ*clpX* strain produced small immature sporangia that contained a much smaller number of spores, whereas the *clpC* mutants produced fragile sporangia that contained a number of spores similar to that of the wild-type strain. Thus, ClpX is involved in sporangium expansion, whereas ClpC is involved in the formation of rigid sporangium structures. Therefore, the target proteins appear to differ between ClpX- and ClpC-containing Clp protease complexes.

Transcriptional and genetic analyses of *clpX* suggested that ClpX is important for both sporangium formation and dehiscence. Gene complementation tests indicated that transcription of *clpX* from either TSS_U_ or TSS_D_ could restore apparently normal sporangium formation in the Δ*clpX* strain, although transcription from the two promoters was activated during sporangium formation (Fig. 3G through J; Fig. 6C). In contrast, sporangium dehiscence required the transcription of *clpX* from both promoters when sporangia were suspended in 25 mM histidine solution, although only FliA-dependent transcription from TSS_D_ was further activated during sporangium dehiscence under this condition (Fig. 4C through E; Fig. 6C). Therefore, the constant transcription of *clpX* from TSS_U_ is required for sporangium dehiscence under this condition. Meanwhile, transcription of *clpX* from either TSS_U_ or TSS_D_ was sufficient for sporangium dehiscence when sporangia were immersed in 25 mM NH_4_HCO_3_ solution on a HAT plate. Although the molecular mechanisms are unknown, we empirically know that the two methods used for inducing sporangium dehiscence have different effects on the efficiency of sporangium dehiscence in some specific mutant strains. For the Δ*clpX* strains harboring *clpX*ΔP_U_ and *clpX*ΔP_D_, overlay of 25 mM NH_4_HCO_3_ solution appeared to create more favorable conditions for sporangium dehiscence than 25 mM histidine solution suspension. In the former method, the eluates from HAT agar may contribute to the favorable conditions. A similar trend was observed in the Δ*clpP3* strain (Fig. 4I; Fig. 5C). Thus, post-translational regulation via proteolysis by the Clp complex may be more important for sporangium dehiscence when sporangia harvested from HAT agar are suspended in 25 mM histidine solution than when 25 mM NH_4_HCO_3_ solution is poured onto the sporangium-forming HAT agar plate, although ClpP3 is expected to be catalytically inactive.

ClpP1 and ClpP4 share the highest sequence similarity (61% identity) among the four proteolytic subunits (Fig. S3B). As described in the Introduction, members of the genus *Streptomyces* encode multiple copies of *clpP* in their genomes. In the phylogenetic tree, the ClpP proteins from *Streptomyces* were found in a clade containing ClpP2, in addition to the clade containing ClpP1 and ClpP4 (Fig. S2B). Therefore, *clpP2* homologs may have occurred in a common ancestor of *Streptomyces* and *Actinoplanes* species. In support of this, homologs of ClpP1, ClpP2, and ClpP4 were found in all 28 *Actinoplanes* species analyzed in this study (Fig. S2B). In contrast, the ClpP3 homologs formed a different clade from the ClpP proteins from *Streptomyces* and were found in only 19 *Actinoplanes* species (Fig. S2B). Therefore, *clpP3* homologs may have occurred within *Actinoplanes* during evolution.

Among the four ClpP proteins in *A. missouriensis*, only ClpP3 does not possess a catalytic triad in its amino acid sequence (Fig. S3A). Therefore, ClpP3 appears to be catalytically inactive. In the unicellular cyanobacterium *Synechococcus elongatus*, a ClpP variant, ClpR, which lacks the catalytic triad, has been reported. In this bacterium, two distinct Clp complexes exist, consisting of a unique proteolytic core comprising two Clp subunits, one with ClpP1 and ClpP2 and the other with ClpP3 and ClpR. The ClpP3/ClpR core consists of twin heptameric rings, each with an identical ClpP3_3_/ClpR_4_ configuration, and is associated with a particular ATPase, ClpC (58, 59). Therefore, we consider that ClpP3 in *A. missouriensis* is an inactive ClpR-type subunit of the Clp complex that forms the proteolytic core together with other active proteolytic subunits. Because the gene disruption experiment revealed that the Δ*clpP3* strain showed a conditional deficiency in sporangium dehiscence, we assume that the Clp complex containing ClpP3 plays a crucial role in sporangium dehiscence induced by the suspension of sporangia in 25 mM histidine solution. Because the sporangia of the Δ*clpP1*, *clpP2* (S113A), and Δ*clpP4* strains normally opened and released spores under the same conditions (Fig. S5), we expect that ClpP3 forms a proteolytic core with at least two different proteolytic subunits and that the distinct Clp complexes compensate for each other during sporangium dehiscence.

Although we failed to obtain the Δ*clpP2* strain by in-frame deletion, the *clpP2* (S113A) mutant strain was successfully generated, which grew normally on YBNM and HAT agar plates and in PYM liquid broth. These results suggest that ClpP2 is required for the viability or normal growth of *A. missouriensis*, but its proteolytic activity is not involved in this physiological function. Alternatively, ClpP2 may not possess proteolytic activity despite the conserved catalytic triad in its amino acid sequence (Fig. S3A). In this respect, it should be noted that three ClpP proteins from *Streptomyces* species, SCO7280 from *S. coelicolor* A3(2), SGR6291 from *Streptomyces griseus*, and Sven0839 from *Streptomyces venezuelae*, do not possess a catalytic triad in their amino acid sequences (Fig. S3C), all of which are found in a clade containing ClpP2 in the phylogenetic tree (Fig. S2B). Moreover, there remains a possibility that ClpP2 is an unusual subunit that exerts proteolytic activity without involving Ser-113. Although further experiments are required to examine whether ClpP2 is catalytically active, ClpP2 seems to be a component of the proteolytic core, together with other catalytically active subunits, such as ClpP1 or ClpP4.

In conclusion, we have demonstrated that functional Clp machinery is required for sporangium formation and dehiscence in *A. missouriensis*. While we have studied the transcriptional regulation of the genes involved in sporangium formation and dehiscence (53, 54, 56, 60, 61), the present study suggests that regulated proteolysis also plays a crucial role in these developmental stages. However, many facets of proteolysis remain unexplored, including the ATPase and proteolytic subunit configuration of the Clp machinery, substrate proteins for hydrolysis, and the physiological functions of the substrates. These challenges for future research will provide further insights into the reproduction processes of *A. missouriensis*.

## MATERIALS AND METHODS

### General methods

Bacterial strains, plasmid vectors, and media used in this study have been described previously (31, 33, 53, 62). Primers used in this study are listed in Table S1. SEM was performed using an S-4800 scanning electron microscope (Hitachi, Tokyo, Japan), as described previously (63). Phase-contrast microscopy was performed using a BH-2 microscope (Olympus, Tokyo, Japan), as described previously (60). The zoospores released from sporangia were counted as described previously (56).

### Construction of mutant strains

To construct the Δ*clpX*, Δ*clpP1*, Δ*clpP3*, and Δ*clpP4* strains, the upstream and downstream regions of the genes were amplified by PCR. The amplified DNA fragments were cloned into pUC19 and sequenced to confirm that no PCR-derived errors were introduced. The cloned fragments were digested and cloned together into pK19mobsacB (64), whose kanamycin resistance gene had been replaced with an apramycin resistance gene (53). Using these plasmids, gene deletion mutant strains were generated using the method described previously (54). Gene disruption was confirmed by PCR. To construct the *clpC* (T30S) and *clpC* (I33F/L34F) mutant strains, the mutated *clpC* gene fragments with upstream and downstream regions were amplified by PCR using genomic DNAs extracted from the T-2 and P-8 strains, respectively (43). The T-2 and P-8 strains are motile suppressor strains obtained from the *trxA* and *ptxA* null mutant strains, respectively (43). The resultant DNA fragments were cloned into pUC19. The generated plasmids were sequenced to confirm that no PCR-derived errors were introduced. The cloned fragments were digested and cloned into pK19mobsacB, whose kanamycin resistance gene had been replaced with an apramycin resistance gene (53, 64). Using these plasmids, *clpC* mutant strains were generated, and the mutations were confirmed by sequencing of the *clpC* gene. To construct the *clpP2* (S113A) mutant strain, the S113A mutation was introduced by overlap extension PCR, and the resultant DNA fragment was cloned into pUC19. The generated plasmid was sequenced to confirm that no PCR-derived errors were introduced. The cloned fragment was digested and cloned into pK19mobsacB, whose kanamycin resistance gene had been replaced with an apramycin resistance gene (53, 64). Using the plasmid, the *clpP2* (S113A) mutant strain was generated, and the mutation was confirmed by sequencing of the *clpP2* gene.

### Construction of strains for the gene complementation test

The 2.1- and 1.2-kbp DNA fragments containing the promoter and coding sequences of *clpX* and *clpP3*, respectively, were amplified by PCR. The amplified fragments were digested with *Hin*dIII and *Xba*I and cloned into pTYM19-Apra (52, 53). The *clpX*ΔP_U_, *clpX*ΔP_D_, and *clpX*ΔP_U_ΔP_D_ genes were amplified by overlap extension PCR and cloned into pTYM19-Apra. The generated plasmids were sequenced to confirm that no PCR-derived errors were introduced and introduced into the Δ*clpX* and Δ*clpP3* strains by conjugation, as described previously (54). Plasmid pTYM19-Apra was also introduced into the wild-type and mutant strains to generate vector control strains. Apramycin-resistant colonies were obtained.

### RNA extraction

Total RNAs were extracted as described previously (53). Briefly, *A. missouriensis* cells were disrupted by rubbing with a mortar and pestle, and the cell lysate was mixed with the lysis/binding solution of the RNAqueous Total RNA Isolation Kit (Thermo Fisher Scientific, MA, USA). After the debris was removed by centrifugation at 20,000 × *g* for 10 min, total RNAs were extracted according to the manufacturer’s instructions. The total RNAs were treated with DNase I to eliminate contaminating genomic DNA and purified by phenol-chloroform extraction and ethanol precipitation.

### S1 nuclease mapping

S1 nuclease mapping was performed as previously described (65, 66). A hybridization probe for the *clpX* transcript was prepared using genomic DNA from the wild-type strain by PCR and ^32^P-labeled at both 5′-ends with [γ-^32^P]ATP (PerkinElmer, MA, USA) using T4 polynucleotide kinase (Takara Biochemicals, Shiga, Japan). ^32^P-labeling at one 5′-end was eliminated by digestion with *Eco*RI. For hybridization, 40 μg of total RNA was used. S1 nuclease-protected fragments were analyzed on a 6% denaturing polyacrylamide-urea gel with size markers. For high-resolution S1 nuclease mapping, protected fragments were analyzed on a 6% denaturing polyacrylamide DNA sequencing gel with sequencing ladders generated according to the method described by Maxam and Gilbert (67).

## SUPPLEMENTAL MATERIAL

Fig. S1 jb.00308-26-s0001.pdf Structure of ClpA from *E. coli* and AlphaFold-based prediction of the structures of five Clp_ATPases from *A. missouriensis*.

Supplemental material jb.00308-26-s0002.pdf Fig. S2 to S5; Table S1.

ASM does not own the copyrights to Supplemental Material that may be linked to, or accessed through, an article. The authors have granted ASM a non-exclusive, world-wide license to publish the Supplemental Material files. Please contact the corresponding author directly for reuse.
