# Supplementary material for: Components of the Clp complex are required for sporangium formation and dehiscence in Actinoplanes missouriensis
Source: J Bacteriol. 2026 Sep 1;208(10):e00308-26. doi: 10.1128/jb.00308-26 (PMC13689560; doi:10.1128/jb.00308-26)
Supplement: Supplementary file 1 [file jb.00308-26-s0001.pdf]

Supplemental Material for

Components of the Clp complex are required for sporangium formation and dehiscence in *Actinoplanes missouriensis*

Ryota Suzuki,[1] Manato Maruko,[1] Remi Suzuki,[1] Takeaki Tezuka,[1,2] Yasuo Ohnishi[1,2]

[a]Department of Biotechnology, Graduate School of Agricultural and Life Sciences, The University of Tokyo, Bunkyo-ku, Tokyo, Japan

[b]Collaborative Research Institute for Innovative Microbiology, The University of Tokyo, Bunkyo-ku, Tokyo, Japan

This PDF file includes:

Figure S1

A ClpA (*E. coli*)

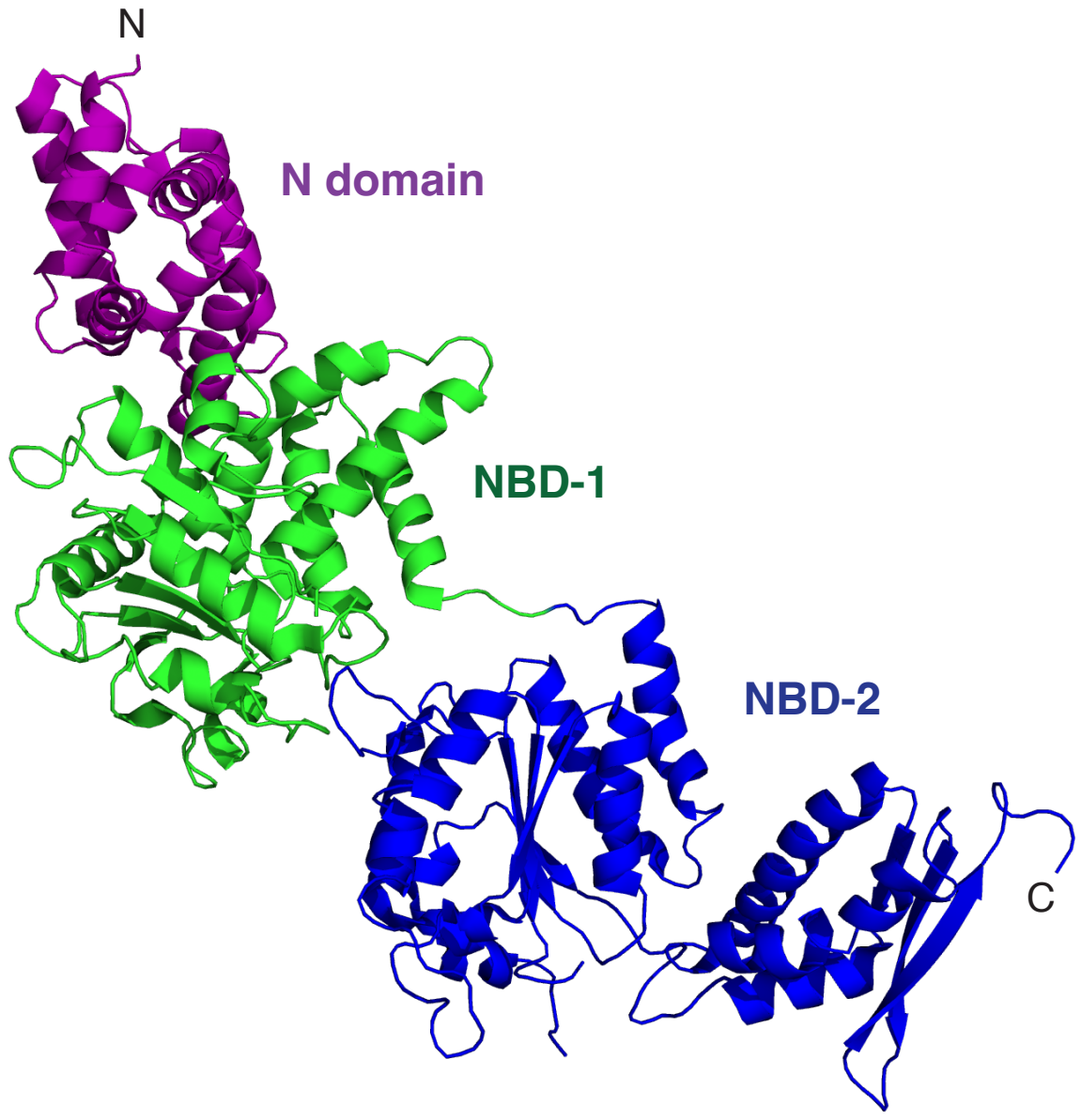

B AMIS_910

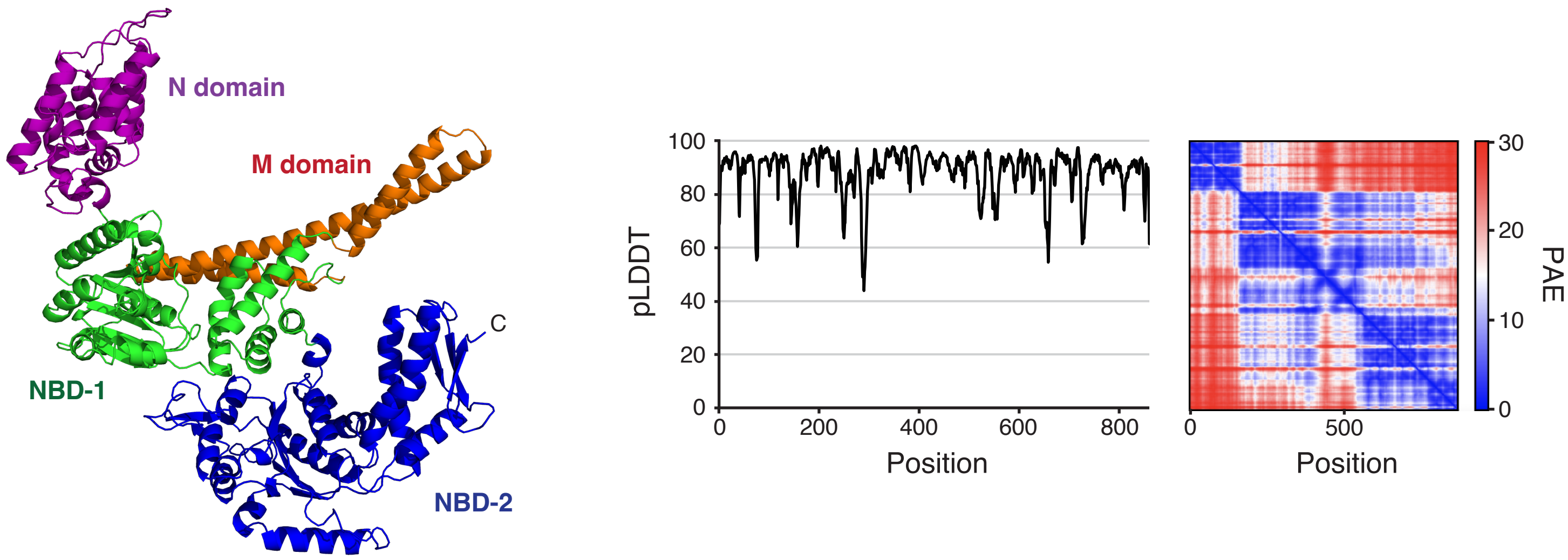

C AMIS_39870

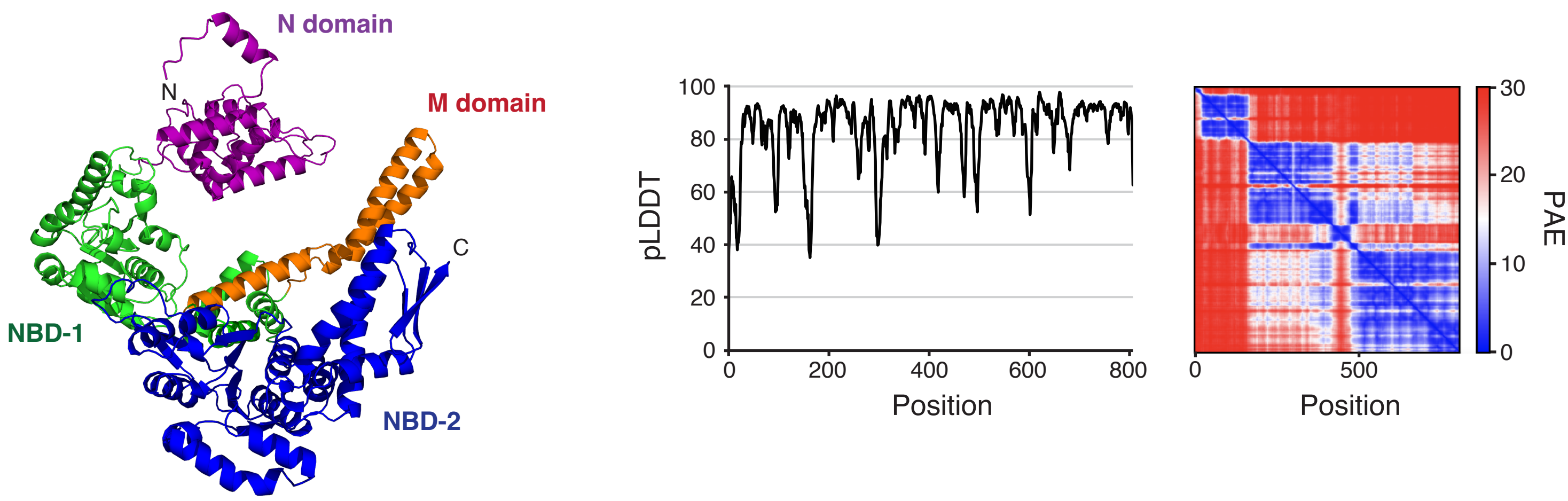

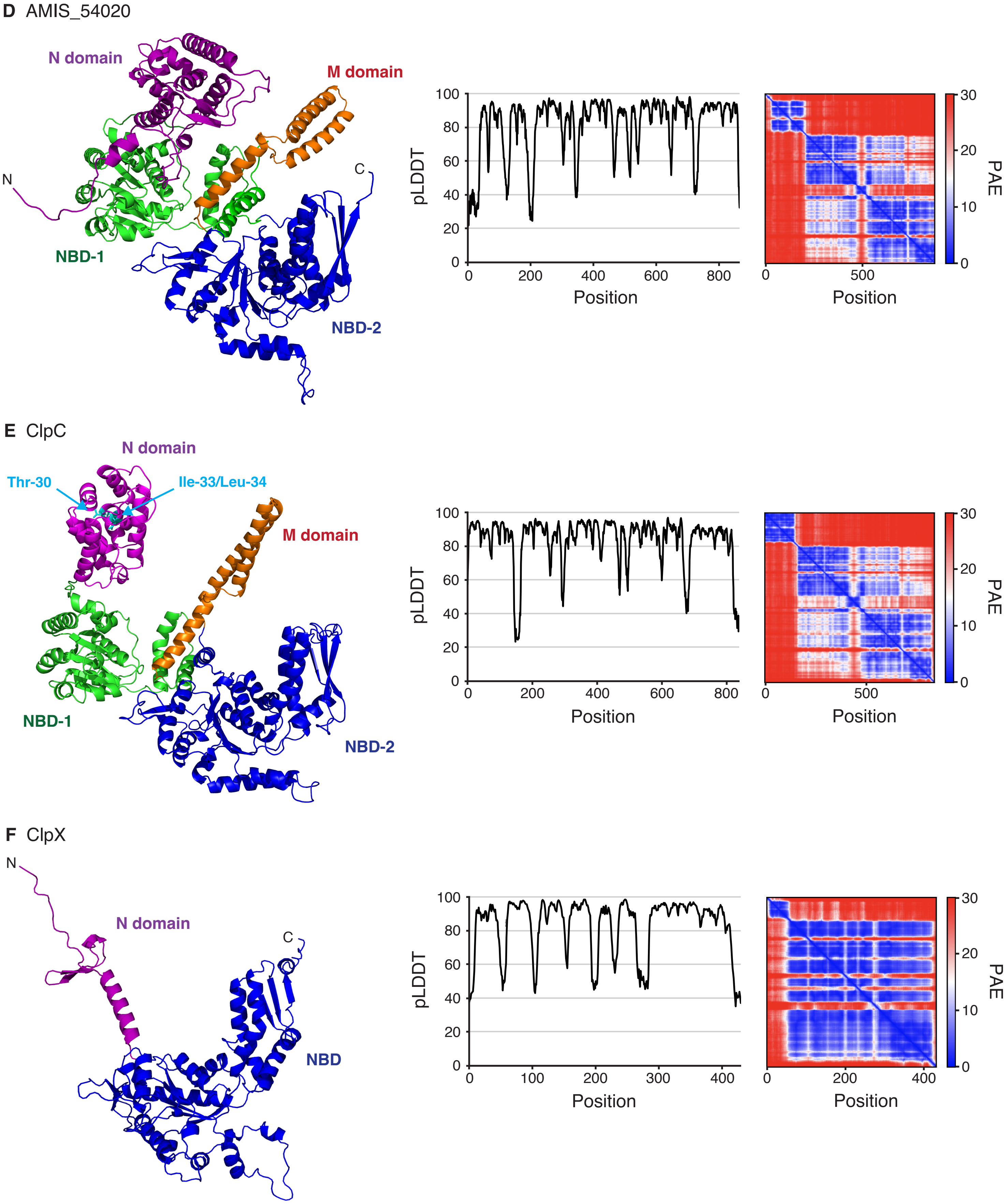

Fig. S1. Structure of ClpA from *E. coli* and AlphaFold-based prediction of the structures of five Clp_ATPases from *A. missouriensis*. Polypeptides are shown by ribbon representation and are colored purple, green, orange, and blue for the N-terminal domain (N domain), first nucleotide-binding domain (NBD-1), middle domain (M domain), and second nucleotide-binding domain (NBD-2), respectively. (A) Protomer structure of *E. coli* ClpA (PDB ID: 1KSF). (B–F) Predicted structures of AMIS_910 (B), AMIS_39870 (C), AMIS_54020 (D), ClpC (E), and ClpX (F). The predicted local distance difference test (pLDDT) score, which characterizes the local structural accuracy, and the predicted aligned error (PAE) score, which corresponds to the topological accuracy between each residue, are shown on the right side of the predicted structures. In (E), the Thr-30, Ile-33, and Leu-34 residues in ClpC are shown in light blue. In (F), the nucleotide-binding domain of ClpX is colored blue because the nucleotide-binding domain of class 2 ATPases is homologous to NBD-2 of class 1 ATPases.
