# Supplementary material for: Components of the Clp complex are required for sporangium formation and dehiscence in Actinoplanes missouriensis
Source: J Bacteriol. 2026 Sep 1;208(10):e00308-26. doi: 10.1128/jb.00308-26 (PMC13689560; doi:10.1128/jb.00308-26)
Supplement: Supplementary file 2 [file jb.00308-26-s0002.pdf]

Supplemental Material for
Components of the Clp complex are required for sporangium formation and dehiscence in *Actinoplanes missouriensis*

Ryota Suzuki,[1] Manato Maruko,[1] Remi Suzuki,[1] Takeaki Tezuka,[1,2] Yasuo Ohnishi[1,2]

[a]Department of Biotechnology, Graduate School of Agricultural and Life Sciences, The University of Tokyo, Bunkyo-ku, Tokyo, Japan

[b]Collaborative Research Institute for Innovative Microbiology, The University of Tokyo, Bunkyo-ku, Tokyo, Japan

This PDF file includes:

Figures S2 to S5
Table S1
Supplemental reference

A

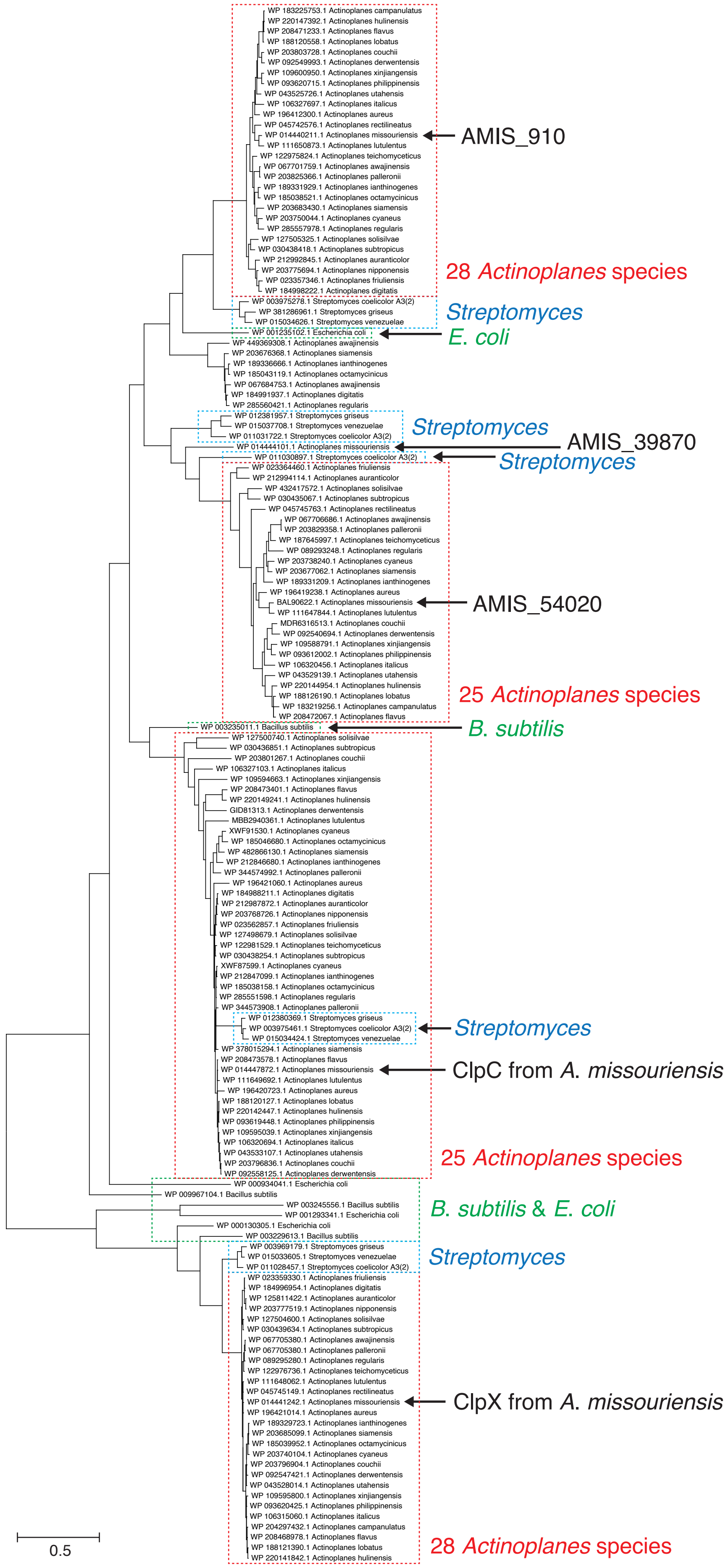

AMIS_910
28 Actinoplanes species
Streptomyces
E. coli
Streptomyces
AMIS_39870
Streptomyces
AMIS_54020
25 Actinoplanes species
B. subtilis
Streptomyces
ClpC from A. missouriensis
25 Actinoplanes species
B. subtilis & E. coli
Streptomyces
ClpX from A. missouriensis
28 Actinoplanes species
0.5

B

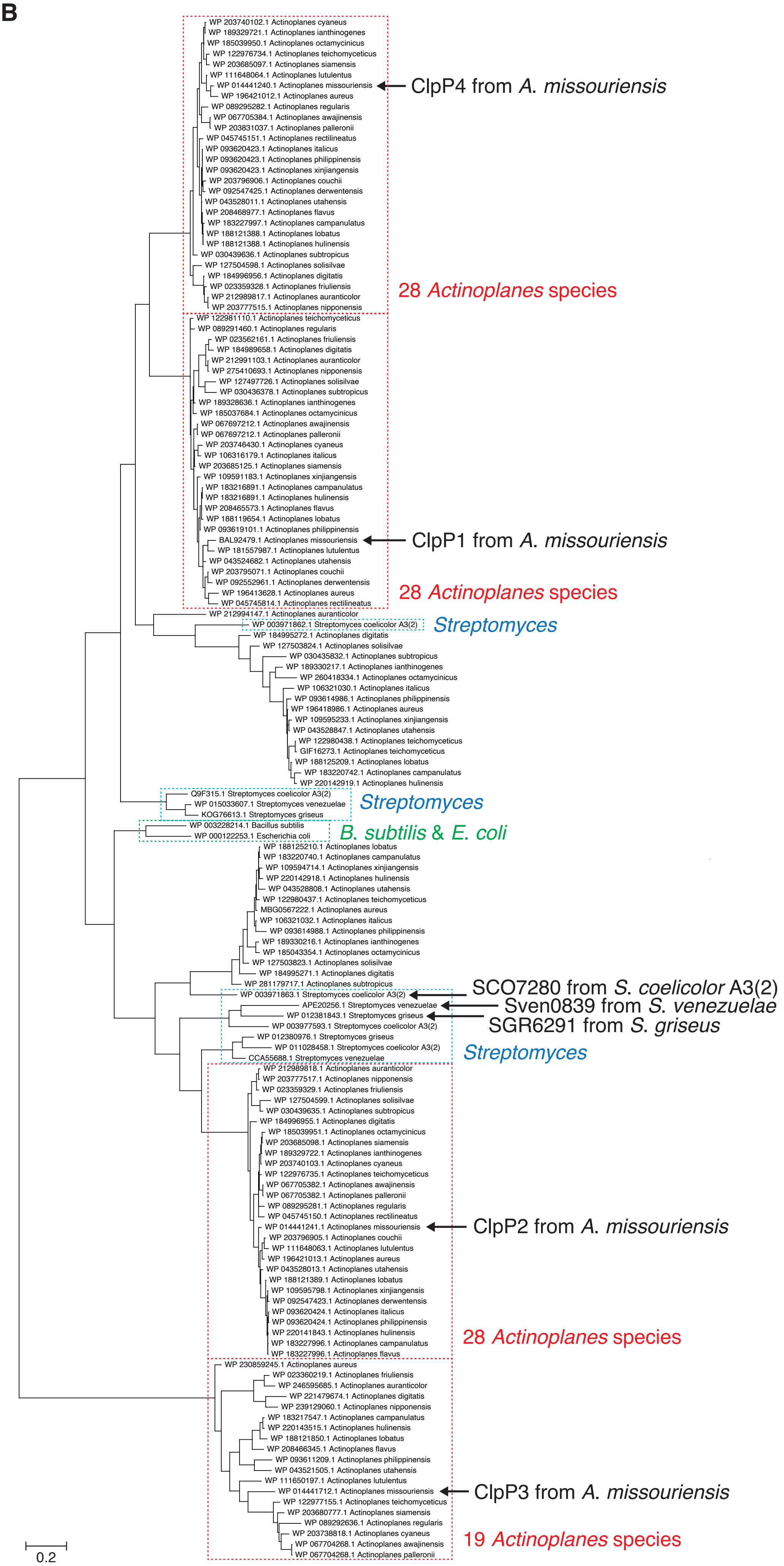
WP 203740102.1 Actinoplanes cyaneus
WP 189329721.1 Actinoplanes ianthinogenes
WP 185039950.1 Actinoplanes octamycinicus
WP 122976734.1 Actinoplanes teichomyceticus
WP 203685097.1 Actinoplanes siamensis
WP 111648064.1 Actinoplanes lutulentus
WP 014441240.1 Actinoplanes missouriensis
WP 196421012.1 Actinoplanes aureus
WP 089295282.1 Actinoplanes regularis
WP 067705384.1 Actinoplanes awajinensis
WP 203831037.1 Actinoplanes palleronii
WP 045745151.1 Actinoplanes rectilineatus
WP 093620423.1 Actinoplanes italicus
WP 093620423.1 Actinoplanes philippinensis
WP 093620423.1 Actinoplanes xinjiangensis
WP 203796906.1 Actinoplanes couchii
WP 092547425.1 Actinoplanes derwentensis
WP 043528011.1 Actinoplanes utahensis
WP 208468977.1 Actinoplanes flavus
WP 183227997.1 Actinoplanes campanulatus
WP 188121388.1 Actinoplanes lobatus
WP 188121388.1 Actinoplanes hulinensis
WP 030439636.1 Actinoplanes subtropicus
WP 127504598.1 Actinoplanes solisilvae
WP 184996956.1 Actinoplanes digitatis
WP 023359328.1 Actinoplanes friuliensis
WP 212989817.1 Actinoplanes auranticolor
WP 203777515.1 Actinoplanes nipponensis
ClpP4 from A. missouriensis
28 Actinoplanes species
WP 122981110.1 Actinoplanes teichomyceticus
WP 089291460.1 Actinoplanes regularis
WP 023562161.1 Actinoplanes friuliensis
WP 184989658.1 Actinoplanes digitatis
WP 212991103.1 Actinoplanes auranticolor
WP 275410693.1 Actinoplanes nipponensis
WP 127497726.1 Actinoplanes solisilvae
WP 030436378.1 Actinoplanes subtropicus
WP 189328636.1 Actinoplanes ianthinogenes
WP 185037684.1 Actinoplanes octamycinicus
WP 067697212.1 Actinoplanes awajinensis
WP 067697212.1 Actinoplanes palleronii
WP 203746430.1 Actinoplanes cyaneus
WP 106316179.1 Actinoplanes italicus
WP 203685125.1 Actinoplanes siamensis
WP 109591183.1 Actinoplanes xinjiangensis
WP 183216891.1 Actinoplanes campanulatus
WP 183216891.1 Actinoplanes hulinensis
WP 208465573.1 Actinoplanes flavus
WP 188119654.1 Actinoplanes lobatus
WP 093619101.1 Actinoplanes philippinensis
BAL92479.1 Actinoplanes missouriensis
WP 181557987.1 Actinoplanes lutulentus
WP 043524682.1 Actinoplanes utahensis
WP 203795071.1 Actinoplanes couchii
WP 092552961.1 Actinoplanes derwentensis
WP 196413628.1 Actinoplanes aureus
WP 045745814.1 Actinoplanes rectilineatus
ClpP1 from A. missouriensis
28 Actinoplanes species
WP 212994147.1 Actinoplanes auranticolor
WP 003971862.1 Streptomyces coelicolor A3(2)
Streptomyces
WP 184995272.1 Actinoplanes digitatis
WP 127503824.1 Actinoplanes solisilvae
WP 030435832.1 Actinoplanes subtropicus
WP 189330217.1 Actinoplanes ianthinogenes
WP 260418334.1 Actinoplanes octamycinicus
WP 106321030.1 Actinoplanes italicus
WP 093614986.1 Actinoplanes philippinensis
WP 196418986.1 Actinoplanes aureus
WP 109595233.1 Actinoplanes xinjiangensis
WP 043528847.1 Actinoplanes utahensis
WP 122980438.1 Actinoplanes teichomyceticus
GIF16273.1 Actinoplanes teichomyceticus
WP 188125209.1 Actinoplanes lobatus
WP 183220742.1 Actinoplanes campanulatus
WP 220142919.1 Actinoplanes hulinensis
Q9F315.1 Streptomyces coelicolor A3(2)
WP 015033607.1 Streptomyces venezuelae
KOG76613.1 Streptomyces griseus
Streptomyces
WP 003228214.1 Bacillus subtilis
WP 000122253.1 Escherichia coli
B. subtilis & E. coli
WP 188125210.1 Actinoplanes lobatus
WP 183220740.1 Actinoplanes campanulatus
WP 109594714.1 Actinoplanes xinjiangensis
WP 220142918.1 Actinoplanes hulinensis
WP 043528808.1 Actinoplanes utahensis
WP 122980437.1 Actinoplanes teichomyceticus
MBG0567222.1 Actinoplanes aureus
WP 106321032.1 Actinoplanes italicus
WP 093614988.1 Actinoplanes philippinensis
WP 189330216.1 Actinoplanes ianthinogenes
WP 185043354.1 Actinoplanes octamycinicus
WP 127503823.1 Actinoplanes solisilvae
WP 184995271.1 Actinoplanes digitatis
WP 281179717.1 Actinoplanes subtropicus
WP 003971863.1 Streptomyces coelicolor A3(2)
APE20256.1 Streptomyces venezuelae
WP 012381843.1 Streptomyces griseus
WP 003977593.1 Streptomyces coelicolor A3(2)
WP 012380976.1 Streptomyces griseus
WP 011028458.1 Streptomyces coelicolor A3(2)
CCA55688.1 Streptomyces venezuelae
SCO7280 from S. coelicolor A3(2)
Sven0839 from S. venezuelae
SGR6291 from S. griseus
Streptomyces
WP 212989818.1 Actinoplanes auranticolor
WP 203777517.1 Actinoplanes nipponensis
WP 023359329.1 Actinoplanes friuliensis
WP 127504599.1 Actinoplanes solisilvae
WP 030439635.1 Actinoplanes subtropicus
WP 184996955.1 Actinoplanes digitatis
WP 185039951.1 Actinoplanes octamycinicus
WP 203685098.1 Actinoplanes siamensis
WP 189329722.1 Actinoplanes ianthinogenes
WP 203740103.1 Actinoplanes cyaneus
WP 122976735.1 Actinoplanes teichomyceticus
WP 067705382.1 Actinoplanes awajinensis
WP 067705382.1 Actinoplanes palleronii
WP 089295281.1 Actinoplanes regularis
WP 045745150.1 Actinoplanes rectilineatus
WP 014441241.1 Actinoplanes missouriensis
WP 203796905.1 Actinoplanes couchii
WP 111648063.1 Actinoplanes lutulentus
WP 196421013.1 Actinoplanes aureus
WP 043528013.1 Actinoplanes utahensis
WP 188121389.1 Actinoplanes lobatus
WP 109595798.1 Actinoplanes xinjiangensis
WP 092547423.1 Actinoplanes derwentensis
WP 093620424.1 Actinoplanes italicus
WP 093620424.1 Actinoplanes philippinensis
WP 220141843.1 Actinoplanes hulinensis
WP 183227996.1 Actinoplanes campanulatus
WP 183227996.1 Actinoplanes flavus
ClpP2 from A. missouriensis
28 Actinoplanes species
WP 230859245.1 Actinoplanes aureus
WP 023360219.1 Actinoplanes friuliensis
WP 246595685.1 Actinoplanes auranticolor
WP 221479674.1 Actinoplanes digitatis
WP 239129060.1 Actinoplanes nipponensis
WP 183217547.1 Actinoplanes campanulatus
WP 220143515.1 Actinoplanes hulinensis
WP 188121850.1 Actinoplanes lobatus
WP 208466345.1 Actinoplanes flavus
WP 093611209.1 Actinoplanes philippinensis
WP 043521505.1 Actinoplanes utahensis
WP 111650197.1 Actinoplanes lutulentus
WP 014441712.1 Actinoplanes missouriensis
WP 122977155.1 Actinoplanes teichomyceticus
WP 203680777.1 Actinoplanes siamensis
WP 089292636.1 Actinoplanes regularis
WP 203738818.1 Actinoplanes cyaneus
WP 067704268.1 Actinoplanes awajinensis
WP 067704268.1 Actinoplanes palleronii
ClpP3 from A. missouriensis
19 Actinoplanes species
0.2

Fig. S2. Phylogenetic relationships of Clp-ATPases (A) and proteolytic subunits (B) of the Clp complex. The maximum likelihood trees were constructed using MEGA X (Kumar *et al*. 2018). A total of 150 (A) and 146 (B) proteins from 28 *Actinoplanes* species, *S. griseus*, *S. coelicolor* A3(2), *S. venezuelae*, *B. subtilis*, and *E. coli* were analyzed. Clades including proteins from *A. missouriensis* are shown in red dotted rectangles. Proteins from *Streptomyces* species are shown in blue dotted rectangles. Proteins from *B. subtilis* and *E. coli* are shown in green dotted rectangles.

A

```
EcClpP  ---MSYSGERDNFAPHMALVPMVIEQTSRGERSFDIYSRLLKERVIFLTGQVEDHMANLIVAQMLFLEAENPEKDIYLYINSP
ClpP1   ----------MTDLHIPATPMARRGEALSGNLDDSVYNRLLRERIIFLGSEVTDAVANRICAQLLLLSAEDPEADIHLWINSP
ClpP2   -MTDLSMPPGFAPVHNRYVLPSFSERTSWGIKESNPYNKMFEDRIIFLGVQVDDASANDVMAQLLTLESTDPDRDILMYINSP
ClpP3   MYNRHDPNRWGFPPHPQPDWQPPHLPGSGPGLPGWLEERLFDQRIVMIRGQLTPDVATGTVAALLTLDSAGPD-PIHLHMASS
ClpP4   ----------MREGGLMSIDTTMRGGGAS--FDDQVFERLLRERIIFLGSEVNDEVTNRICAQMLLLASEDSERDITLYINSP

EcClpP  GGVITAGMSIYDTMQFIKPDVSTICMGQAASMGAFLLTAGAKGKRFCLPNSRVMIHQPL-GGYQGQATDIEIHAREILKVKGR
ClpP1   GGSVYSGMAIYDTMQFIDNDVSTVGMGMAASMGQFLLCAGTAGKRYALPHARIMMHQPS-GGMGGTAADIAIQAEQMLYTKKM
ClpP2   GGSFTAMTAIYDTMQYVRPDISTVCLGQAASAAAVLLAGGTPGKRMALPHSRIIIHQPATEGGYGQGSDIEIQAREIMRMRTQ
ClpP3   GGDLSAALSIIDVIDSLTAPVQALVTSEAG--GAVLAVLAASDQRSAYRHARFKLAEPRAAGVTGTADEVAAAAGQHLRELEE
ClpP4   GGSISAGMAVYDTMQYIKNDVATVAMGMAASMGQFLLTAGTAGKRFALPHARVMMHQLS-GGIGGTAADIAIQAESMLHIKQV
```

Ser-113 in ClpP2

```
EcClpP  MNELMALHTGQSLEQIERDTERDRFLSAPEAVEYGLVDSILTHRN----------
ClpP1   FQERISYHTGQSPEQIAADFDRDRWFTAAEAKDYGFIDKVITGATQVPDGAGTLN
ClpP2   LEDMIAHHSNQSVEKVRKDIDRDKIMTADEAKEYGLVDVVLATRKKSLQAVGASN
ClpP3   VVVRLAEVTGQPRSRVEDDLSTGRTLSATEALEYGLITEVITPKPR---------
ClpP4   MNERIAFHSGHTPEEIERDSDRDRWFTAPQAKDYGLVDHVITTTADVHTGASIV-
```

B

	ClpP2	ClpP3	ClpP4
ClpP1	44%	26%	61%
ClpP2	–	23%	40%
ClpP3	–	–	22%

C

```
EcClpP    ---------------------MSYSGERDNFAPHMALVPMVIEQTSRGE-RSFDIYSRLLKERVIFLTGQVEDHMANLIVAQM
SCO1238   ------------------------------MTRPSARHVLPEFTERTSAGTRTSDPYSKLLQERIVFLGTPVDETSANDVTAQL
SCO2618   MNDFPGSGLYDRVNAAQDMRAASQGRYTGPQAESRYVIPRFVERTSQGVREYDPYAKLFEERVIFLGVQIDDASANDVMAQL
SCO2619   -------------------MRRPGAVVRRAGGYVTNLMPSAAGEPSIGGGLGDQVYNRLLGERIIFLGQPVDDDIANKITAQL
SCO7280   ----------------------------------MGSYTIPNVVERTPQGERSYDVFSRLLSERIIFLGTEIDDGVANVVIAQL
SCO7281   -------------------------MSPFTAGPAPARTPRAEEGDTPATRFDDRLAEQLLDQRIVLLGTQVDEVSANRVCAQL
SGR4923   ----------------------------------MTNLMPYAAGEPSLGGGLGDQVYSRLLGERIIFLGQQVDDDIANKITAQL
SGR4924   ---------MVKTHMNNYTGASASGLYTGPQVDNRYVVPRFVERTSQGVREYDPYAKLFEERVIFLGVQIDDASANDVMAQL
SGR6291   ------------------------------MIRPAARYVLPEFTERTATGTRTLDPYSKLLSERIVFLGSPIDDIAASDLIAQL
Sven0839  ---------------------------MEVPAMDARHVLPAFTERTSYGTRTLDPYSKLLESRIVFLGTPIDETAANDVIAQL
Sven2402  ----------------MQNNLSPSGLYTGAPMDNRYVVPRFVERTSQGVREYDPYAKLFEERVIFLGVQIDDASANDVMAQL
Sven2403  ----------------------------------MTNLKPYAAGEPSIGGGLGDHVYNRLLGERIIFLGQQVDDEIANKITAQM

EcClpP    LFLEAENPEKDIYLYINSPGGVITAGMSIYDTMQFIKPDVSTICMGQAASMGAFLLTAGAKGKRFCLPNSRVMIHQP--LGG
SCO1238   MYLEHQAPDRDIELYVNSPGGSFTAMTAIYDTMRYVACDVATTCLGQAGPSAAVLLAAGTPGKRAALPGARVVLHQPALTEP
SCO2618   LCLESMDPDRDISVYINSPGGSFTALTAIYDTMQYVKPDVQTVCMGQAASAAAVLLAAGTPGKRMALPNARVLIHQPYSET-
SCO2619   LLLAS-DPDKDIFLYINSPGGSITAGMAIYDTMQYIKNDVVTIAMGLAASMGQFLLSAGTPGKRFALPNAEILIHQP--SAG
SCO7280   LHLESSAPESEIAVYINSPGGSFTSLMAIYDTMTFVQAPISTFCVGQAASTAAVLLAGGDPGRRFVLEHARVLLGQPASGG-
SCO7281   LILSAQDPRTDISLYVNSPGGSVHAGLAIYDTMRLIPNDVSTLAMGFAASMGQFLLSVGTAGKRYALPNARIMMHQP--SAG
SGR4923   LLLAA-EPDKDIYLYINSPGGSVTAGMAVYDTMQYIPNDVVTIGMGMAASMGQFLLTGGAAGKRFALPNTDILMHQG--SAG
SGR4924   LCLESMDPDRDISIYINSPGGSFTALTAIYDTMQFVKPDIQTVCMGQAASAAAVLLAAGTPGKRMALPHARVLIHQPSSQT-
SGR6291   MYLEHADPDRPLSLYINSPGGTFQAMAAVYDTMRFLTCEVETFCLGQAGSYAAALLAGGTKGRRHALPGARVVIQQPAMEEP
Sven0839  LYLDHAGPDQDIALYINSPGGSVSAMTAIYDTMGSLHCDLETTCLGQAMSTAAVLLAAGTPGKRMMLPGARVTLQEPAMDEP
Sven2402  LCLESMDPDRDISIYINSPGGSFTALTAIYDTMQFVKPDIQTVCMGQAASAAAVLLAAGTPGKRMALPNARVLIHQPSGGT-
Sven2403  LLLAA-DPDKDIYLYINSPGGSVTAGMAVYDTMQYIPNDVVTIGMGMAASMGQFLLTGGTPGKRFALPNTDILMHQG--SAG

EcClpP    YQGQATDIEIHAREILKVKGRMNELMALHTGQSLEQIERDTERDRFLSAPEAVEYGLVDSILTHRN-------------
SCO1238   VRGQAGDLAVHAAELVRVRARLEEILVRHTGRTPGQVAADLERDTVLDARQAREYGLVDRIVPGRRTPPASSGAR----
SCO2618   GRGQVSDLEIAANEILRMRSQLEEMLAKHSTTPVEKIREDIERDKILTAEDALSYGLIDQIITTRKMDNSSLR------
SCO2619   LAGSASDIKIHAERLLHTKRRMAELTSQHTGQTIEQITRDSDRDRWFDAFEAKEYGLIDDVIATAAGMPGGGGTGA---
SCO7280   RQGTVSDLALQAKEMVRIRSQVEEVLARHTHHDVATLRADMDRDKVFTAQEAVAYGLADEVLARRLTRV----------
SCO7281   IGGTTADIEIQADNLDFTKRTIERITAEHTGQSPETISRDGDRDRWFTAEEAREYGMVDQVVQSLADVRPAATRRRMGL
SGR4923   IGGTASDIKIQAQYLLRTKTRMAEITAHHSGQTVETIIRDGDRDRWYTAEEAKDYGLIDEIITVASGIPGGGGTGA---
SGR4924   GREQLSDLEIAANEILRMRTQLEQMLARHSTTPLEKISEDIERDKILTAEDALAYGLVDQIVSTRKTTAGASL------
SGR6291   MRGQPSDLEIHARELVRTREMFAAMLVRDTGRTAEQITADIERDTILDAEAALAHGLVDHVVANR--------------
Sven0839  LRGQPSDLDLQAREMLRLRALMAGMLTAHTGRDRERIDADLDRLTVLDAPAALAYGLVDHVITSRRGPAGGAAG-----
Sven2402  GREQLSDLEIAAREILRMRTQLEDMLAKHSSTPIEKIRDDIERDKILTAEDALAYGLVDQIVSTRKSTAAAAH------
Sven2403  IGGTASDIKIQAEYLLRTKQRMAEITAHHSGQTVEAIIRDGDRDRWFTADEAKEYGLIDDIITHAAGVPGGGGTGA---
```

Fig. S3. Sequence similarity of ClpP proteins. (A) Amino acid sequence alignment of ClpP from *E. coli* (*Ec*ClpP) and ClpP1, ClpP2, ClpP3, and ClpP4 from *A. missouriensis*. The residues comprising the catalytic triads are indicated by red asterisks. Three aromatic residues in *Ec*ClpP, Tyr-60, Tyr-62, and Phe-82, which comprise the hydrophobic surface, are shown with blue asterisks. Ser-113 in ClpP2 is indicated by a red arrowhead. (B) Amino acid sequence identities among ClpP1, ClpP2, ClpP3, and ClpP4 in *A. missouriensis*. (C) Amino acid sequence alignment of *Ec*ClpP, five ClpP proteins (SCO1238, SCO2618, SCO2619, SCO7280, and SCO7281) from *S. coelicolor* A3(2), three ClpP proteins (SGR4923, SGR4924, and SGR6291) from *S. griseus*, and three ClpP proteins (Sven0839, Sven2402, and Sven2403) from *S. venezuelae*. The residues comprising the catalytic triads are indicated by red asterisks. The three aromatic residues in *Ec*ClpP are indicated by blue asterisks.

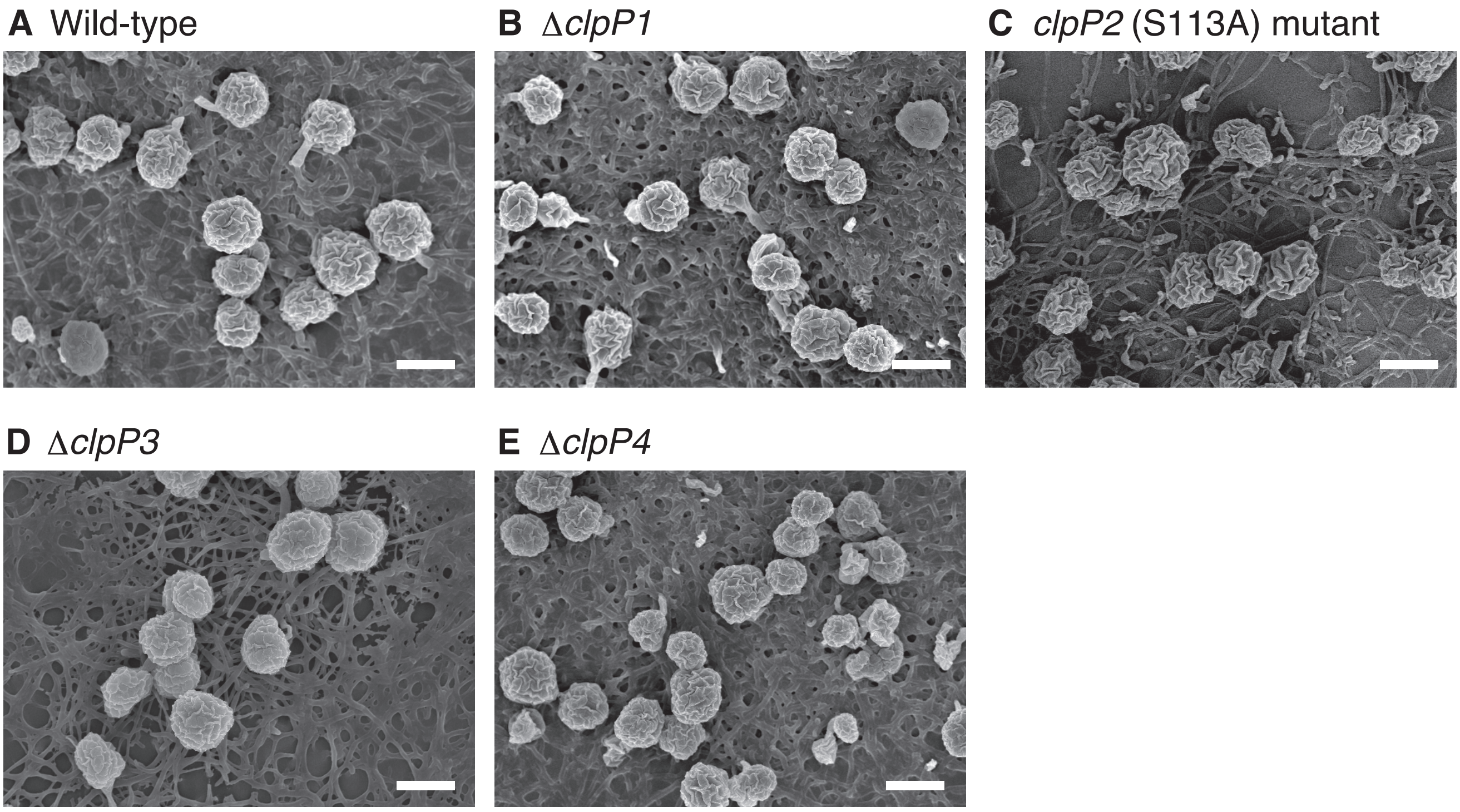

Fig. S4. SEM analysis of sporangia and mycelia produced on HAT agar after 7 days of cultivation. (A) The wild-type strain. (B) The Δ*clpP1* strain. (C) The *clpP2* (S113A) mutant strain. (D) The Δ*clpP3* strain. (E) The Δ*clpP4* strain. Bars, 5 μm.

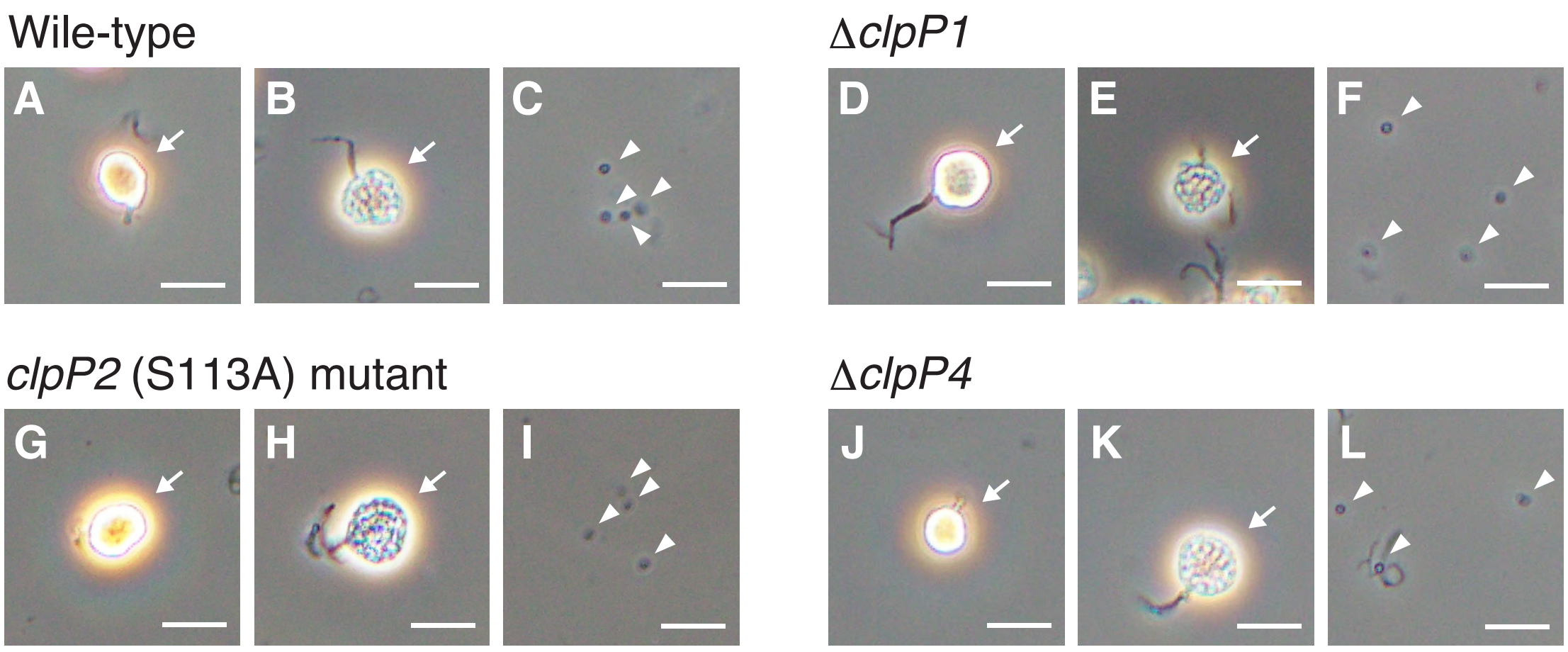

Fig. S5. Observation of sporangium dehiscence by phase-contrast microscopy. Sporangia produced on HAT agar were harvested and suspended in 25 mM histidine solution to induce sporangium dehiscence. Micrographs of the wild-type strain (A–C), Δ*clpP1* strain (D–F), *clpP2* (S113A) mutant strain (G–I), and Δ*clpP4* strain (J–L) are shown. Panels A, D, G, and J were taken immediately after suspension. Panels B, E, H, and K were taken 15 min after suspension. Panels C, F, I, and L were taken 30 min after suspension. Sporangia (including those with transparent envelopes) and released spores are indicated by arrows and arrowheads, respectively. Bars, 10 μm.

Table S1. Primers used in this study

Primer name	Sequence (5' to 3')[a]	Used for
AMIS_11250-UF	GGAATTCGAGAAGACCGAGGAGCAGAT	Gene disruption
AMIS_11250-UR	GCTCTAGACTTCAGTAGGTCGCCACCGT	Gene disruption
AMIS_11250-DF	GCTCTAGAAACGTGAACCCGACGATCGT	Gene disruption
AMIS_11250-DR	ACATGCATGCGCATGTGTTAAGCACGCCGC	Gene disruption
AMIS_11230-UF	GGAATTCAGAACGCCGCAAGATCCACA	Gene disruption
AMIS_11230-UR	GCTCTAGAGTAGACCGAATCGTCGAGGT	Gene disruption
AMIS_11230-DF	GCTCTAGAATCGACAAGGTGATCACCGG	Gene disruption
AMIS_11230-DR	CCCAAGCTTGTCGAGCTCGAAGAGACGCT	Gene disruption
AMIS_15950-UF	GGAATTCGGTTGAGCCCTTCGTAGAGA	Gene disruption
AMIS_15950-UR	GCTCTAGACAGGTGCGGAGGTTGCCAGT	Gene disruption
AMIS_15950-DF	GCTCTAGACTCGAGTACGGGCTGATCAC	Gene disruption
AMIS_15950-DR	CCCAAGCTTTCAGGATGATCGGCAGCTTG	Gene disruption
AMIS_72590-UF	GGAATTCACAAGCGTGATGAACGCCGC	Gene disruption
AMIS-72590-UR	GCTCTAGACCGCTCGAACACCTGGTCGT	Gene disruption
AMIS_72590-DF	GCTCTAGAGACCATGTGATCACCACGAC	Gene disruption
AMIS_72590-DR	CCCAAGCTTAACTGGTCGTGCACGAACGC	Gene disruption
S_t-2/S_t-7/S_{1780}-8_ClpC-F1	GGAATTCAGAATCCAGCAGGAAGTCGA	Mutant construction
S_t-2/S_t-7/S_{1780}-8_ClpC-R1	CCCAAGCTTCTCACTCCTCGGTAGTGGCA	Mutant construction
AMIS_11240_S113A_F1	GGAATTCTCACAACCACTGATCTT	Mutant construction
AMIS_11240_S113A_F2	CCAGGCGGCCGCCGCCGCGGCT	Mutant construction
AMIS_11240_S113A_R1	AGCCGCGGCGGCGGCCGCCTGG	Mutant construction
AMIS_11240_S113A_R2	CCCAAGCTTACCTCGTCGATGTAGATGAT	Mutant construction
cAMIS_11250-F1	CCCAAGCTTACCGGATCATCTTCCTCGGC	Gene complementation
cAMIS_11250-R1	GCTCTAGAACCGGAAAGAACTGCTACGC	Gene complementation
cAMIS_11250-P_U-F2	CCACAGCGGGCGAGTCTGCAGCCTCAGCTA	Gene complementation
cAMIS_11250-P_U-R1	TGCAGACTCGCCCGCTGGTGGGGCTGTTCTC	Gene complementation
cAMIS_11250-P_D-F2	CGGTTCAGCGGAGATGGCAACGGGGCAATCG	Gene complementation
cAMIS_11250-P_D-R1	TTGCCATCTCCGCTGAACCGCGTAGCTGAG	Gene complementation
cAMIS_15950-F1	CCCAAGCTTGGCCCGGAGCCGCACGACGTC	Gene complementation
cAMIS_15950-R1	GCTCTAGACTGAAGCGCCTAGGAGAACG	Gene complementation
clpX_S1-F	GGAATTCACCCCTGACACGCCCGTTTT	S1 nuclease mapping
clpX_S1-R	AGGAGCACTTCAGTAGGTCG	S1 nuclease mapping
clpX_P1-F	GGAATTCAAGAAGAGCCTGCAGGCCGT	S1 nuclease mapping
clpX_P1-R	GTCCGTGTCCGAGGCGGATT	S1 nuclease mapping
clpX_P2-F	GGAATTCAGTAACTGAGCGCGGTGTCA	S1 nuclease mapping
clpX_P2-R	TTCTCCCTGCGTTTCATGGC	S1 nuclease mapping

[a]The recognition sequences for restriction enzymes are underlined.

Supplemental reference

Kumar S, Stecher G, Li M, Knyaz C, Tamura K. 2018. MEGA X: Molecular evolutionary genetics analysis across computing platforms. *Mol Biol Evol* **35**:1547–1549.
